# Automated DNA mutation detection using universal conditions direct sequencing: application to ten muscular dystrophy genes

**DOI:** 10.1186/1471-2156-10-66

**Published:** 2009-10-18

**Authors:** Richard R Bennett, Hal E Schneider, Elicia Estrella, Stephanie Burgess, Andrew S Cheng, Caitlin Barrett, Va Lip, Poh San Lai, Yiping Shen, Bai-Lin Wu, Basil T Darras, Alan H Beggs, Louis M Kunkel

**Affiliations:** 1 Program in Genomics and Division of Genetics, and The Manton Center for Orphan Disease Research, Children's Hospital Boston, Boston, Massachusetts, USA; 2 Department of Laboratory Medicine, Children's Hospital Boston, Boston, Massachusetts, USA, and Department of Pathology, Harvard Medical School, Boston, Massachusetts, USA; 3 Department of Paediatrics, Yong Loo Lin School of Medicine, National University of Singapore, Singapore; 4 Department of Neurology, Children's Hospital Boston, Boston, Massachusetts, USA; 5 Department of Pediatrics, Harvard Medical School, Boston, Massachusetts, USA; 6 Department of Genetics, Harvard Medical School, Boston, Massachusetts, USA; 7 Howard Hughes Medical Institute, Children's Hospital Boston, Boston, Massachusetts, USA, and Harvard Medical School, Boston, Massachusetts, USA

## Abstract

**Background:**

One of the most common and efficient methods for detecting mutations in genes is PCR amplification followed by direct sequencing. Until recently, the process of designing PCR assays has been to focus on individual assay parameters rather than concentrating on matching conditions for a set of assays. Primers for each individual assay were selected based on location and sequence concerns. The two primer sequences were then iteratively adjusted to make the individual assays work properly. This generally resulted in groups of assays with different annealing temperatures that required the use of multiple thermal cyclers or multiple passes in a single thermal cycler making diagnostic testing time-consuming, laborious and expensive.

These factors have severely hampered diagnostic testing services, leaving many families without an answer for the exact cause of a familial genetic disease. A search of GeneTests for sequencing analysis of the entire coding sequence for genes that are known to cause muscular dystrophies returns only a small list of laboratories that perform comprehensive gene panels.

The hypothesis for the study was that a complete set of universal assays can be designed to amplify and sequence any gene or family of genes using computer aided design tools. If true, this would allow automation and optimization of the mutation detection process resulting in reduced cost and increased throughput.

**Results:**

An automated process has been developed for the detection of deletions, duplications/insertions and point mutations in any gene or family of genes and has been applied to ten genes known to bear mutations that cause muscular dystrophy: DMD; CAV3; CAPN3; FKRP; TRIM32; LMNA; SGCA; SGCB; SGCG; SGCD. Using this process, mutations have been found in five DMD patients and four LGMD patients (one in the FKRP gene, one in the CAV3 gene, and two likely causative heterozygous pairs of variations in the CAPN3 gene of two other patients). Methods and assay sequences are reported in this paper.

**Conclusion:**

This automated process allows laboratories to discover DNA variations in a short time and at low cost.

## Background

This study focused on ten muscular dystrophy genes described in Online Mendelian Inheritance in Man [1], (Table 1); however the resulting process can be applied to any gene or family of genes.

**Table 1:** OMIM and Accession numbers

	*Homo Sapiens*					
**Gene**	**OMIM#**	**Genomic RefSeq**	**NM_#**	v.*	**Entrez** **Gene ID**	**UniProt ID**

DMD	300377	NC_000023	004006	.2	1756	P11532

CAV3	601253	NG_008797	033337	.1	859	P56539

CAPN3	114240	NG_008660	000070	.2	825	P20807

TRIM32	602290	NG_011619	012210	.3	22954	Q13049

FKRP	606596	NG_008898	024301	.4	79147	Q9H9S5

LMNA	150330	NG_008692	170707	.2	4000	P02545

SGCA	600119	NG_008889	000023	.2	6442	Q16586

SGCB	600900	NG_008891	000232	.4	6443	Q16585

SGCG	608896	NG_008758	000231	.2	6445	Q13326

SGCD	601411	NG_008693	000337	.5	6444	Q92629

**O**MIM = Online Mendelian Inheritance in Man, NM_# are the RefSeq numbers for the most common muscle isoform mRNA.* version is the latest NM_# version at time of publication and also the version used for CDS nucleotide numbering in Table 7 HGVS variation nomenclature

There are over 40 primary congenital muscle disorders (Additional file 1) determined now more by the defective genes causing the disorders rather than specific clinical descriptions. Many of these diseases are rare. The incidence, defined as the number of new cases per million live births, (or alternatively as the fraction of live births represented by one new case) is not known for many of the more rare diseases; nevertheless, these primary muscle disorders can be subdivided into three groups in which estimates of incidence based on published data can be deduced (Table 2).

**Table 2:** Incidence data for three subdivisions of primary muscle disorders

	Maximum incidence		Minimum incidence		Average	
**Type**	**freq. × 10^-6^**	**fraction**	**freq. × 10^-6^**	**fraction**	**×10^-6^**	**fraction**

DMD/BMD	354	1/2825	186	1/5376	270	1/3703

Other dystrophies	100	1/10000	41	1/24390	70	1/14286

Congenital myopathies	219	1/4566	100	1/10000	160	1/6250

*Total*	673	1/1486	327	1/3050	500	1/2000

DMD mean incidence is well established at 300 × 10^-6 ^(Emery 2002) but data varies from country to country and survey to survey. It is unlikely to be much higher but may be as low as 169 × 10^-6 ^(Cowan 1980). BMD frequency is based on (Emery 1991). Thankfully, both DMD and BMD incidence is probably declining annually due to genetic testing and counselling.

Other dystrophy incidence numbers are scarce to non-existent. The rough estimate maximum is extrapolated from (Bushby 2001) and minimum from (Emery 2002).

Other congenital myopathy numbers are also scarce to non-existent. The maximum estimate is extrapolated from (Nonaka 2001) as 81% of the mean DMD incidence. The minimum estimate is extrapolated from (D'Amico 2008) and (Lopate 2007).

These data were used for estimation of testing cost and should be considered as rough estimates only.

The most common form of muscular dystrophy is X-linked Duchenne muscular dystrophy (DMD) with approximately 1 in 4200 newborn males affected per year worldwide [2-5]. Allelic to DMD, the milder form called Becker muscular dystrophy (BMD) is the second most common form of the disease. Together DMD and BMD affect approximately 1 in 3600 newborn males per year worldwide [4] and compose nearly 80% of all new cases of dystrophy and 56% of all new cases of congenital myopathy of all types. Female carriers of DMD/BMD are born with approximately the same frequency as male patients [6,7]. The underlying cause of DMD and BMD was identified over 20 years ago [8-10] as abnormalities of the DMD gene, encoding the protein named dystrophin.

Approximately 60% of DMD and BMD cases are caused by large deletions (one exon or greater) or large duplications in the DMD gene [11,12]. In the past, when a new patient was suspected of having some type of muscular dystrophy, the first molecular test prescribed was the Chamberlain-Beggs multiplex PCR test [12,13]. This test detected 98% of large deletions and some duplications in the dystrophin gene. The test is composed of four groups of multiplexed PCR assays covering 19 hot spot exons out of the 79 exons of the DMD gene. Sometimes when the test detected one or more deleted or duplicated exons, additional adjacent exons not in the hot spot region had to be tested separately in order to find the break points which are important for differentiating BMD from DMD. This test has now been replaced in most laboratories by the Multiplex Ligation-Dependent Probe Amplification (MLPA) test [14] for aberrant copy number in the Duchenne muscular dystrophy gene. MLPA tests all 79 exons in two reactions, (P034-DMD-1 and P035-DMD-2, MRC-Holland; Willem Schoutenstraat 6; 1057 DN Amsterdam; The Netherlands) and will detect the exact molecular cause, including break points, for approximately 60% of DMD and BMD cases.

Various forms of direct sequencing and exon pre-screening from patient DNA have been used to detect the majority of the remaining 40% of mutations in the DMD gene. These remaining mutations are mostly small (less than one exon) variations collectively known as point mutations [15] including insertions, duplications, deletions, deletions plus insertions (indels) and single or multiple base changes. One of the most common and efficient methods for detecting mutations in the exons and regions of interest in disease genes is Polymerase Chain Reaction (PCR) amplification of the area of interest resulting in millions of copies of that DNA fragment followed by direct sequencing of the fragment. Until 2003, and even now, in many laboratories, the process of designing PCR assays (flanking sense and anti-sense primers to amplify the regions of interest in DNA) has been to focus on the individual PCRs and not on the complete set of PCRs to test a gene or a group of genes. Primers for each individual PCR were selected primarily based on location and sequence concerns. Melting temperatures for the two primers were calculated, followed by attempts to adjust the primer sequences to nearly match each other's melting temperature. Finally, annealing temperature for the PCR was set approximately 5°C below the primer Tm's [16]. This process generally results in groups of assays with varying annealing temperatures requiring either the use of multiple thermal cyclers or multiple passes in a given thermal cycler. This makes diagnostic testing nearly impossible to automate and therefore time-consuming, laborious and expensive.

In 2003, a methodology termed Single Condition Amplification/Internal Primer (SCAIP) [17] was introduced and applied to the DMD gene superceding previous methodologies such as Detection Of Virtually All Mutations (DOVAM) [18] and Denaturing High Performance Liquid Chromatography (DHPLC) [19]. The major advantage of SCAIP is that all assays are performed at a single set of thermal cycler conditions in a single multi-well plate. Among the other advantages of SCAIP is that the use of internal sequencing primers provides increased specificity of each assay to the area of DNA under examination. As published, the SCAIP method uses a series of ethanol washes for PCR and cycle sequencing reaction purification and it uses original phred/phrap/consed software [20] for sequence analysis. One potential drawback to the SCAIP method is that if a single amplicon does not amplify, possibly due to polymorphic sequence at the primer site, the assay must be repeated using various combinations of the PCR and internal sequencing primers. At the time of publication, SCAIP was applied to the DMD gene only. Many other forms of congenital myopathies remain with little or no options for diagnostics. A search of GeneTests [21] for sequencing analysis of the entire coding sequence for other myopathy genes aside from DMD returns a small number of choices at fairly high costs and long turn-around times. A partial list of available services, costs and turn-around time (TAT) is summarized in tables 3&4 and Additional file 2. Therefore in many families a molecular diagnosis may be difficult or impossible to obtain due to the lack of relevant tests, high cost, or long turn-around time of existing tests. This serves as motivation for all clinicians and researchers to offer ever more accurate and fast diagnostic testing of these diseases that is offered at low cost to affected families. There is progress in that direction. In 2006, Tjeldhorn et al. demonstrated a reduction from 72 hours manual labor in the fully manual mutation detection processing for 16 Marfan syndrome patients to 23 hours of manual work in an automated process reducing testing time from 145 total hours to 67 hours[22]. In addition in 2008, protocols were published for mutation detection using automated fluorescence-based sequencing[23,24]. With more than 40 different primary muscle disorders, a different approach to sequencing and diagnostics was desperately needed. Early in 2003, this study began in order to determine if a more automated and less costly mutation detection process could be developed expanding the SCAIP concepts to encompass more genes and also to more fully automate the process. By taking advantage of some modern computer aided design tools, the most recent database updates, and SNP tools, primer sequences were designed that avoided common simple nucleotide polymorphisms. In theory, eliminating polymorphisms from primer sequences enhances the detection of subsequent heterozygous polymorphisms (important for autosomal disorders) and limits the number of assays that need to be re-amplified using different primers. Due to more careful designing of primers and avoidance of simple nucleotide polymorphisms, the technique of using universal sequencing primers appended to the 5' end of the PCR primers was enabled. The major benefit of universal sequencing tails is that it not only enables universal sequencing reaction conditions for all assays, but also avoids the necessity of separate internal sequencing primers simplifying the task of automation. This automation could be accomplished by taking advantage of improvements in purification techniques (e.g. magnetic beads), more user friendly sequence analysis tools [e.g. Mutation Surveyor™ (SoftGenetics, LLC. State College, PA), Seqscape™ (Applied Biosystems Inc. part of Life Technologies, Carlsbad, CA), Sequencher™ (Gene Codes Inc., Ann Arbor, MI)], newly available DNA analyzers [e.g. the QIAxcel™ system (formerly eGene system, QIAGEN Inc., Valencia CA)] and robotics [e.g. Hamilton MicroLab StarPlus™ (Hamilton Co., Reno, NV)].

**Table 3:** Survey of services available for DMD/BMD point mutations

Laboratory	Pricing	TAT	comments
Athena Dx Worcester, MA	$5070.00	6-8 weeks	

City of Hope Los Angeles, CA	$1860.00	10 weeks	

Emory Univ. Atlanta, GA	$2313.00	4-6 weeks	Resequencing array

CHB*	$3634.00	6-8 weeks	

U. of Utah	$1175.00	6 weeks	Negative -del/dup testing required

Data gathered from GeneTests http://www.genetests.org and telephone survey of available services for DMD/BMD point mutation testing. *Children's Hospital Boston current testing based on DHPLC method, UCDS not yet priced

(Note that the survey was done in April, 2009 and the services fortunately are constantly being expanded and improved.)

**Table 4:** Survey of services available for LGMD gene mutations

Laboratory	Gene Panel	Genes Tested	Pricing	TAT
Athena Dx Worcester, MA	Yes (9 genes)	CAPN3, SG's, DYSF, CAV3, FKRP, LMNA	$10,790.00 (total)	5-6 weeks

Nationwide Children's Ohio State	No	CAPN3, SG's, DYSF, CAV3, FKRP, LMNA	$874.50-$1790.75 (per gene) (varies by size)	4 weeks

Prevention Genetics Marshfield, WI	No	CAPN3, DYSF, FKRP, LAMA2, SGCA, SGCB (6 genes singly sequenced)	$390.00-$2990.00 (per gene) (varies by size)	40 days/gene

U. of Utah	Yes (2 genes)	DYSF & CAPN3	$1500.00 (total)	8 weeks

U of Iowa	Yes (3 genes) (panel)*	SGCA, SGCB, FKRP CAPN3, LMNA, FKRP, POMT1, POMT2, POMGnT, FCMD**	$905panel* $462-1781 (per gene) (varies by size)	2-4 wks

Data gathered from GeneTests http://www.genetests.org and telephone survey of available services for LGMD gene testing. * The U of Iowa panel tests for three known mutations only, if none is positive then the entire FKRP gene is sequenced. **7 genes singly sequenced. (Note that the survey was done in April, 2009 and the services fortunately are constantly being expanded and improved.)

Thus a process could be nearly fully automated such that a technician running the process would only have to prepare master mixes and transport plates between equipment stations in the process. At the conclusion of this study, at least ten muscular dystrophy genes have one or more automated mutation detection methods available. The study's results however can be applied to genes involved in any inherited disease or trait. Here we report on the development of the process including assay primer sequences for ten muscular dystrophy genes (Additional files 3, 4, 5, 6, 7, 8, 9, 10, 11 and 12), Hamilton MicroLab StarPlus automated methods (Additional files 13 and 14), Seqscape templates (Additional files 15, 16, 17, 18, 19, 20, 21, 22, 23, 24, 25, 26 and 27), PCR recipes and conditions, and patient mutations found.

## Results

### Primer design

Primers were designed using computer aided design tools such as OLIGO™ (Molecular Biology Insights, Inc., Cascade, CO), and by using the ever improving BLAST and SNP features of public and private databases such as SNPbrowser™ [25], NCBI dbSNP [26], Ensembl [27], Lieden [28], NCBI/BLAST[29], UCSC [30], HapMap [31] and others. Amplicon size was targeted for 600 bases for best sequencing results but this was not always possible due to exon size, polynucleotide strings greater than 7 bases or GC rich clusters greater than 5 bases which do not sequence well. Assays could be designed for all regions of interest in most genes with enough specificity and lack of common polymorphisms within the primer sequences to avoid the necessity of internal primers and to allow for the use of universal sequencing primers appended 5' to the PCR primers. This resulted in assays (including a universal forward and reverse sequencing tail) associated with a gene or gene family that would all amplify and sequence efficiently at a universal set of conditions. In some instances, in collaboration with Applied Biosystems Inc., primers from the VariantSeqR™ (Applied Biosystems Inc. part of Life Technologies, Carlsbad, CA) product line were validated The sequences of VariantSeqR assays are available on the NCBI web page [32]. Simultaneously, automation tools and methods were evaluated or developed (Additional files 28, 29, 30, 31, 32, 33, 34, 35 and 36).

### Amplification optimization

**Figure 1:**
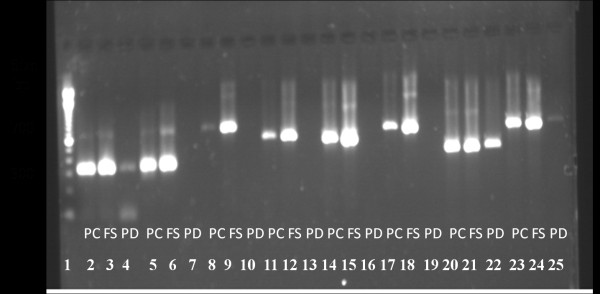
**Polymerase trial experiments**. lane 1 DNA ladder, lanes 2-4 are DMD assay #1 lane 2 polymerase C (PC), lane 3 Roche FastStart (FS), lane 4 polymerase D (PD) lanes5-7 are DMD assay #1a lane 5 PC, lane 6 FS, lane 7 PD, lanes 8-10 are DMD assay #1b lane 8 PC, lane 9 FS, lane 10 PD, lanes 11-13 are DMD assay #2 lane 11 PC, lane 12 FS, lane 13 PD, lanes 14-16 are DMD assay #4 lane 14 PC, lane 15 FS, lane 16 PD, lanes 17-19 are DMD assay #10 lane 17 PC, lane 18 FS, lane 19 PD, lanes 20-22 are FKRP assay #24 lane 20 PC, lane 21 FS, lane 22 PD, lanes 23-25 are FKRP assay #21 lane 23 PC, lane 24 FS, lane 25 PD.

Robust amplification was achieved after multiple experiments with PCR polymerases, plate seals, 384 well PCR plates, and automated liquid handlers from various vendors. PCR polymerase kits from five vendors were tested to determine which kit would represent the highest probability of success over a wide range of target sequence variability. Two vendor polymerase kits failed in early experiments due to weak bands having less than 2.0 ng/uL of product DNA (data not shown). Three more were then tested including polymerase C, Roche FastStart (FS) and polymerase D against a subset of 8 assays (Figure 1).

Roche FastStart efficiently amplified all eight assays and was chosen for all future experiments. Early in the study there was significant evaporation from wells of the 384-well plates probably due to inefficient seals that were being used at the time. After experimenting with BioRad MSB1001 and Applied Biosystems 4306311 the results indicated that neither allowed well contents to evaporate when carefully applied using an adhesive film applicator (Applied Biosystems part # 4333183). Choice of PCR plate is important both for success of amplification and for compatibility with automation equipment. After experiments with plates from a few vendors, Thermo Fisher Scientific Inc. part #AB1111 plates were chosen for our automated process. After much investigation of liquid handling systems, Hamilton MicroLab StarPlus was chosen for the process. Results have been very consistent as measured by spot checking amounts pipetted. After using agarose gels and other DNA analysis systems, the QIAxcel, an automated 12-channel capillary electrophoresis system, was chosen for deletion/duplication detection and for PCR product quantification due to its automation of the process, superior accuracy and ease of use. PCR reaction volumes of 24, 12, 6, and 3 uL were compared over 8 assays. No reactions worked at 3 uL, but all worked at 6 uL though some were below design goal concentrations of PCR product (data not shown). The 12 uL reaction volume was chosen for our automated process because we wanted the smallest volume possible in order to reduce costs but were not yet confident enough in the 6 uL volume.

### Assay optimization and verification

A total of 309 assays spanning ten known muscular dystrophy causing genes have been designed and/or verified to date and are laid out in three plates (Table 5).

**Table 5:** Genes covered to date

Plate	Protein	Phenotype	# exons	# Assays
				
Genes				
PLATE #1				

DMD	dystrophin	Duchenne/Becker	79	128

PLATE #2				

CAV3	caveolin	LGMD 1C	2	11

FKRP	fukutin related	LGMD 2I(CMD)	4	18

CAPN3	calpain3	LGMD 2A	30	52

TRIM32	tripartate contain32	LGMD 2H	3	15

PLATE #3				

SGCA	sarcoglycan alpha	LGMD 2D	10	13

SGCB	sarcoglycan beta	LGMD 2E	6	20

SGCG	sarcoglycan gamma	LGMD 2C	8	20

SGCD	sarcoglycan delta	LGMD 2F	10	17

LMNA	lamin a/c	LGMD 1B(EDMD)	12	15

Lists the genes covered by plates 1 through 3, the proteins coded by those genes, the associated phenotype when the gene is mutated, the number of exons and assays associated with each gene.

**Figure 2:**
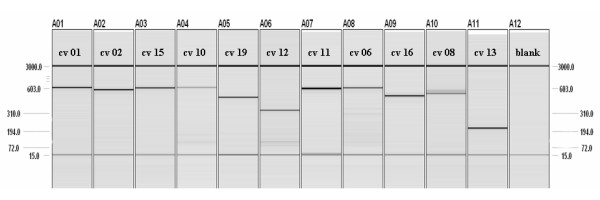
**QIAxcel gel image CAV3 assays**. Columns 1 through 11 contain PCR products from control DNA for CAV3 assays. The 15 base pair and 3000 base pair bands are reference markers injected before the PCR products are injected. Note that the 46XY control DNA that was used may be heterozygous for assay cv 08. Single bands and good forward sequence for this assay in several patients' DNA has been seen. Also there is a large CA repeat in the area which renders the reverse sequence poor. See Additional file 8 for size, concentration and gene coverage details.

All 309 assays were verified by performing PCR using unaffected control DNA, analyzing the products of PCR using the QIAxcel, and by sequencing those products in the ABI 3730 DNA Analyzer™ sequencer (Applied Biosystems Inc. part of Life Technologies, Carlsbad, CA). Assay design was an iterative process. For example, of 45 assays designed in-house, 35 were successful on first trial and did not require redesign (78% success on first trial), and of the 10 remaining which were redesigned, eight were successful on their first trial and did not require additional redesign (80% success on first trial of these 10 redesigned primers). The final two unsuccessful assays were redesigned again, and both were successful (100% success for these last two designs). Therefore, even when using computer aided design tools, the process of designing successful assays is still iterative. An example of QIAxcel data is presented in Figure 2 and Table 6 for the CAV3 gene and in Additional files 37, 38, 39 and 40 for the DMD gene.

**Table 6:** QIAxcel spreadsheet of size and concentration data

Assay Name	CV01	CV02	CV15	CV10	CV19	CV12
size (bp)	621.3	581.4	605.9	627.7	489.3	332.4

conc. (ng/uL)	8.13	8.07	7.05	2.77	6.7	5.49

Assay Name	CV11	CV06	CV16	CV08	CV13	

size (bp)	594.7	609.5	507.3	537/566	208.9	

conc. (ng/uL)	18.97	6.77	9.02	4.64/2.78	13.35	

Compare to Figure 2: QIAxcel gel image CAV3 assays

### Sequence quality and detection of heterozygosity

Sequence data from both the sense and anti-sense direction of each amplicon (the sequence between the two PCR primers of the assay) was analyzed using the software application Mutation Surveyor and/or SeqScape and/or Sequencher. All three provide quality scores for each base read using a proprietary algorithm developed starting from original phred/phrap/consed software. As an example of typical quality of sequence, for the 2490 bases of coding region of the CAPN3 gene for patient 1092.1, 2401 bases had quality scores above 25 (range 0-50) (96.4%), 82 bases had quality scores between 15 and 25 (3.3%) and 7 bases had quality scores below 15 (0.3%). Sensitivity in detection of autosomal heterozygosity is illustrated in Additional file 41: Figures One, Two, Three and Four. The detection of a heterozygous deletion/insertion mutation requires that the reviewer must be vigilant in recognizing and deciphering when the sequence is high quality (that is clean separated peaks for each base with low background noise and high quality scores) up to a point from left to right and also high quality [or in the particular case of Additional file 41: Figure Three, acceptable quality (i.e. although the quality scores are lower for each peak and the background noise is higher, each peak is still separate and well above background)] up to the same point from right to left. This is usually indicative of an insertion (or duplication) or deletion often of just one or a few bases on one allele only. This situation is illustrated in Additional file 41: Figures Three and Four for patient 1102.1. The analyst should also use simple nucleotide polymorphism information to help determine heterozygosity of each allele. As can be seen in Figure One of Brockington et al[33], heterozygous variations can either be detected or easily overlooked.

Using assays that meet the goals specified in the Methods section, five suspected DMD patients who had tested negative for large deletions or duplications in tier 1 testing (MLPA) and 13 additional patients with muscular dystrophies of unknown etiology were tested. Mutations were found in the DMD gene of the five suspected DMD patients and in four non-DMD/BMD patients with an initial diagnosis of limb-girdle muscular dystrophy (LGMD) of unknown etiology including the following: one FKRP, one CAV3, and two likely causative pairs of variations in the CAPN3 gene of two patients (Table 7).

**Table 7:** Patient samples tested to date and variations found

Plate #	Patient #	Gene	Variation: DNA experimentally determined(protein: theoretically deduced)	Exon/Intron	CK value	Age of Onset	DYS -IHC
1	B1101.1	DMD	c [2521C>T] (p.Gln841X)	20	2601	Female->10 y/o	N/A

1	9	DMD	c.8038C>T(p.Arg2680X)	55	11,010	8	N/A

1	174	DMD	c.2614_2615insA (p.?)	20	N/A	6	N/A

1	343	DMD	c.829C>T(p.Gln277X)	8	32,000	5	N/A

1	383 (BMD)	DMD	c.13208_13209ins ACCTTATGTGACGCTGG	3'UTR	3,935	11	N/A

1	B1105.1	DMD	no causative variation found		1500	18	

1	B1028.1	DMD	no causative variation found		10,000	5	absent

1	1	DMD	no causative variation found		N/A	N/A	N/A

1	B646.1	DMD	no causative variation found		450	20's	

2	B646.1	FKRP	c [826C>A]+ [826C>A](p.Leu276>Ile)	4	450	20's	

2	B1141.1	CAV3	c [84C>A]+ [=](p.Asp28Glu)	1	2400	37	Normal

2	B1092.1	CAPN3	c [551C>T(+)706G>A] (p.Thr184Met +Ala236Thr)	4,5	289	6	Normal

2	B1102.1	CAPN3	c [550delA(+)1967dupACATTTTCAAGCAG]	4,17	2000	34	faint

2	B1103.1		no causative variation found		9505	1	Normal

2	B112.1		no causative variation found		156	1	Normal

2	B1149.1		no causative variation found		1074	10	

2	B122.1		no causative variation found		593	2	Normal

2	B695.1		no causative variation found		321	2-3	Normal

*2*	B848.1		no causative variation found				Normal

Far left column lists the plate number used to test the sample, the next column lists patient sample number, the next column lists the gene in which a causative or possibly causative variation was found and the next column lists variations. N/A means data not available. Note that patient samples with no causative variation found on one plate may be repeated on another plate, and that patients have not yet been tested against all three plates.

### Costs and Turn-Around Time

Reagent costs will vary in near direct proportion to PCR and sequencing reaction sizes. The goal is to reduce these reaction sizes to as small as possible while remaining consistent with sequence and gene coverage goals. Table 8 in the methods section presents an estimate of reagent and plastic (consumables) costs based on 12 uL PCR and 10 uL sequencing reaction volumes. The resulting consumables cost per patient for plate 1 is $532.00 and for plate 2 is $395.00. One approach to pricing the service would be to average the price per patient over a typical mix of 100 muscular dystrophy patients across all five tiers of testing. Using this approach the resulting averaged price obtained from the calculations in the methods section for a typical patient sample is $1040.00 for direct labor plus consumables.

**Table 8:** Estimated reagent and sequencing costs

Reagent	Cost/well	Extended cost Plate 1	Extended cost Plate 2 or 3
Assay	$0.03		

Fast Start	$0.34		

dNTPs	$0.04		

10× Buffer	$0.02		

Sub Total	$0.43		

X6/5*		$0.52	

X8/7#			$0.49

Plastic ware	$0.06	$0.06	$0.06

QIAxcel system	$0.25	$0.25	$0.25

AmPure	$0.07	$0.07	$0.07

Total PCR reagents		$0.90/patient PCR	$0.87/patient PCR

Big Dye	$0.90	$1.80/patient PCR^Θ^	$1.80/patient PCR

Buffer	$0.06	$0.12/patient PCR	$0.12/patient PCR

Plate	$0.01	$0.02/patient PCR	$0.02/patient PCR

CleanSeq	$0.30	$0.60/patient well	$0.60/patient PCR

Plate	$0.01	$0.02/patient PCR	$0.02/patient PCR

Total per patient well		$3.46	$3.43

Total per patient		$442.88	$329.28

*Add ~20% for waste*		$532.00	$395.00

* for one negative control per 5 patients on plate 1; # one negative control per 7 patients on plates 2 or 3; ^Θ ^for both Forward and Reverse sequence/PCR.

20% waste value is a conservative estimate of waste established over many years and used by our clinical and research labs in calculating material waste. It includes remaining reagents in large cocktail batches and possible manual repeats.

The sample processing labor hours estimate (16 hours per patient), also provided in the methods section, for each tier of tiers 2-4 is conservative for steady state continuous processing with dedicated equipment including one automated liquid handler, two 96 capillary sequencers, three QIAxcel systems and four 384-well PCR blocks.

Turn-around time for a particular individual would be less than one week in steady state continuous processing mode. However, in stop and start mode with tasks other than sample processing interleaved, the turn-around time would be much longer.

## Discussion

By optimizing the primer design, reaction conditions and automation of the process, a cost effective and sensitive approach to sequencing of multiple genes involved in an overlapping set of muscle degenerative disorders has been developed. Results of experiments done during this study show that not all polymerases, plate sealing films, multi-well plates, liquid handling systems, PCR reaction volumes and DNA analysis systems are created equal, and that care must be taken in choosing or changing any of these variables as they can have a major impact on successful implementation as well as cost. However, the principle question to be answered by this study was whether or not the use of up to date human genome information including simple nucleotide polymorphisms available in various databases and computer aided design tools would allow the design of specific assays that would PCR amplify and cycle sequence at a universal set of conditions. Note that it is wise when designing primers for PCR assays, especially for amplification of regions of interest in the human genome, to take into account simple nucleotide polymorphisms which include Single Nucleotide Polymorphism (SNPs), as well as Deletion/Insertion Polymorphism (DIPs or INDELs), Short Tandem Repeat (microsatellite) Polymorphism (STRs), and Multiple Nucleotide Polymorphism (MNPs). This is true because if the chromosome/patient being assayed is homozygous for the minor allele, the primers may not bind as well, which, depending on the location of the polymorphism within the primer may decrease the efficiency and sensitivity of the assay either minimally or severely. Of greater concern when screening for heterozygous mutations in autosomal disorders or genotyping is that if the patient/chromosome is heterozygous, the primer will hybridize more efficiently to the strand carrying the major allele and produce allele specific amplification results. Subsequent sequencing or genotyping will then fail to detect heterozygous bases within the amplicon and may cause the reviewer to falsely rule out a candidate gene or mutation. Data from this study indicates that a nearly fully automated and less costly mutation detection process that was developed for ten genes has the capability to encompass many more genes, and that internal sequencing primers (although they do increase specificity) will not be necessary if sufficient care is taken in designing assays. Internal primers instead of universal sequencing primers, however, would not be incompatible with this process.

The data also supports the concept that using computer aided design tools such as Oligo and NCBI/Blast to find primers that meet all the design criteria of this automated process (Additional file 42) will increase the percentage of successful assays that are sensitive to autosomal heterozygosity from roughly 50% attained by simply drawing a line under 19-24 bases on target sequence (estimate based on previous experience) to greater than 80%.

The full answer to the question of how effective our automated process is at finding mutations in patients will only be evident after a large number of patients have been tested. To date the data provides a reasonable degree of optimism and has been recognized as sufficient validation to commence use as the clinical process at Children's Hospital Boston. Cost and throughput analysis done in this study supports the conclusion that the automated process described herein is a more cost effective and higher throughput method of mutation detection than manual direct sequencing.

### Troubleshooting amplification and sequencing failures

Regardless of how carefully assays are designed there are bound to be certain combinations of patient DNA and assay that will fail to amplify or sequence properly even when the patient DNA is not deleted or duplicated in the area being amplified. If this is anything but a rare occurrence for a given assay, the assay must be redesigned. For those rare occurrences, many troubleshooting techniques do exist. For example, one can retry the assay manually (on the bench) using the same primers as well as alternative primers, including positive and negative control templates. Also many PCR kits contain alternative reagents for GC rich and AT rich regions of DNA and there is a large body of literature available for optimization of PCR and sequencing reactions which may need to be investigated in rare situations. For example, 1 M final concentration Betaine and/or 5% final DMSO may be included into the PCR recipe to assist the polymerase in GC rich sequence areas. Since this kind of manual troubleshooting will negatively impact TAT and cost, it should be done in the research and development mode. Only robust well tested assays should be used in the clinical setting. For occasional rare failures, after a first round of troubleshooting in the clinical department, the problem assay should be assigned to the research department to resolve.

### Gross and single exon alterations in autosomal genes

Although the spectrum of mutations in the DMD gene is well established as approximately 60% large alterations (one exon or greater) and 40% point mutations, it is far less well established in the remaining nine genes addressed in this manuscript. A brief review of mutations found in these genes in the Leiden muscular dystrophy databases and the literature leads one to believe that the percentage of large alterations in these genes is less than 15%[34]. Therefore we will test for these types of mutations in tier 5 in patients who have not had mutations identified in tiers 1 through 4. Tier 5 will consist of MRC-Holland art. nr. P176-CAPN3, P116-SGC, P048-LMNA/MYOT, and P268-DYSF (though we have not developed point mutation assays for DYSF). Although simple nucleotide polymorphisms are to be avoided in primer design, they are beneficial in other portions of the amplicon as they may assist the analyst in determining the homozygous, heterozygous or hemizygous status of a particular allele. Availability of parental and sibling DNA will of course be especially valuable in this regard.

### Implications and the importance of mutation detection

Determining an exact molecular diagnosis is important for numerous reasons including family planning, carrier status of extended family, and diagnosis and treatment in other family members. Physicians can more accurately determine a prognosis when the type of muscular dystrophy and molecular cause is known. Many potential therapy trials involve mutation specific treatment and require a molecular diagnosis in order to be eligible for participation. Finally for many patients and families there is a sense of discontent from not knowing a definitive diagnosis of their disease.

### Automation of the process and Alternatives to the process

All of the pipetting steps of the process can be accomplished manually in either 384-well or 96-well plates using a multi-channel pipettor. However, manually pipetting into a 384-well plate can be quite tedious and prone to mistakes while using a 96-well plate probably would require increasing the PCR reaction volume from 12 μL to 18 μL. Either of these manual approaches would forfeit the benefits of automation. Suggested layouts for 384-well plates for tiers 2-4 are available in previously mentioned additional files. (Note that tier 1 is simply the running of the MLPA test for DMD large deletions or duplications). However, any layout that works in a given lab setting including testing of a single gene at a time is fine and will require only minor modifications to the Hamilton MicroLab StarPlus methods written for the suggested layouts. The process we have developed is flexible enough to allow for improvements. Many alternatives exist for automating the process and the choice of the proper equipment is crucial to successful implementation. For a list of suggested evaluation criteria see Additional file 43.

Alternatives to our process are on the horizon as well. For example, next (or second) generation sequencing [massively parallel sequencing by synthesis (using probe ligation, single-base extension or pyrosequencing technology)] promises to be capable of sequencing whole chromosomes or targeted areas on multiple chromosomes in a single sequencing run in the near future.

Also CustomSeq™ Resequencing arrays (Affymetrix Inc. Santa Clara, CA) offer promise as possibly another alternative to come. When this study began in 2003, there were problems with this technology and it was decided that the probability of achieving full coverage of each gene at quality goals specified and at low enough cost per patient was lower at that time than our process. In the future, with technological improvements, resequencing arrays may be a better alternative. Emory University Genetics Laboratory is currently offering both CGH array and resequencing array for the DMD gene. A new project involving DNA-chip arrays is beginning at the TREAT-NMD project team, at the Institute of Human Genetics, University of Newcastle upon Tyne, United Kingdom and may prove a future alternative.

## Conclusion

Successful assays can be designed using computer aided design tools which will all amplify at a single set of thermal cycler conditions and sequence at a single set of cycle sequencing conditions. This enables many assays to be robotically assembled into a single 384-well plate and cycled at a universal set of conditions. Due to the automation and the smaller volumes of the automated process described herein, the costs and turn-around time of diagnostic testing can be reduced significantly from high current costs and long lag times, especially for the limb-girdle muscular dystrophies.

Diagnostic laboratories around the world can now use an automated process to identify mutations in the ten genes addressed in this study. This process can be applied using techniques described in this paper, the referenced papers and the additional files to many other genes as well, including the remaining muscle disease causing genes and genes involved in other disease families with multiple causative genes (e.g. Fanconi Anemia and other bone marrow failure anemias, Charcot-Marie Tooth Neuropathies, etc.).

## Methods

### Assay primers design

Assay primers were designed for all regions of interest using Oligo, NCBI/SNPdb, NCBI/BLAST, Ensembl, and other computer aided design tools as well as SeqScape and Sequencher for an optimum annealing temperature of 58-60°.

### Assay storage and distribution to 384-well plates

Primers were received from the vendor in a 2.0 mL vial in a lyophilized state with the antisense (Coding, Forward or Upper) primer and the sense (Non-coding, Reverse or Lower) primer in separate tubes. Primers were suspended to 60 μM in warm 25% glycerol and 75% nuclease free de-ionized water. From these stocks, working assays were prepared in 1.4 mL ScreenMate™ tubes (PN 4247) with SepraSeal™ caps PN 4464 (Matrix Technology Corp. Hudson, N.H.) using 960 μL of 25% glycerol and 20 μL of forward primer 60 μM stock and 20 μL of reverse primer 60 μM stock. This resulted in a solution of 1.2 μM each or 2.4 μM total primer concentration in 25% glycerol. The Hamilton MicroLab StarPlus distributed 6 μL of this assay solution into the wells of a 384-well plate and the plate was dried (70°C for 40 min.) to 1.5 μL of pure glycerol at 4.8 μM each or 9.6 μM total primer concentration. Similarly, the M13F (5'-TGTAAAACGACGGCCAGT-3') and M13R (5'-CAGGAAACAGCTATGACC-3') primers were ordered and received in 2.0 mL vials in a lyophilized state in separate tubes and suspended to 60 μM in warm 25% glycerol and 75% nuclease free de-ionized water. From these stocks working concentrations at 1.8 μM in 25% glycerol were prepared. All stocks in 25% glycerol can be stored for short term at 4°C if tightly capped (and perhaps wrapped in parafilm) and at -20°C if stored for longer than 4 weeks. The Hamilton MicroLab StarPlus distributed 2 μL of forward or reverse sequencing primer into all wells of a 384-well plate (one plate for M13F and one plate for M13R), and the plates were dried (70°C for 20 min.) to 0.5 μL of pure glycerol at 7.2 μM concentration. All plates that contain primers diluted in pure glycerol may be sealed and stored at room temperature for future use or used immediately. 1.5 μL of glycerol will not adversely affect a 12 μL total volume PCR, nor will 0.5 μL of glycerol adversely affect a 10 μL total volume cycle sequencing reaction.

### PCR reaction assembly

PCR reactions were assembled in a 384-well plate (AB1111, Thermo Fisher Scientific Inc., Waltham, MA) automatically using a Hamilton MicroLab StarPlus liquid handling/pipetting system.

### DNA extraction and distribution to 384-well plates

DNA was extracted and purified from saliva, blood or tissue according to standard protocol from vendors (e.g. Oragene™, DNA Genotek Inc., Ottawa, Ontario, Canada; Gentra Puregene™, QIAGEN Inc. Valencia, CA). As currently configured, 16.0 μg is required to complete all five tiers of testing. Genomic DNA was diluted first to 100 ng/μL in nuclease free ddH_2_O and then to 10 ng/μL using 1× PCR buffer.

The Hamilton MicroLab StarPlus distributed 3 μL of sample DNA to the appropriate wells of the 384-well PCR plate that already contains assays in 1.5 μL glycerol (Additional files 44 and 45).

### Master Mix preparation and distribution

A master mix was prepared consisting of 5.17 μL ddH_2_O (nuclease free), 0.93 μL 10× buffer (0.5 M Tris HCl, 0.1 M KCl, 0.05 M (NH_4_)_2_SO_4 _and 0.0208 M MgCl_2 _PH 8.1), 1.2 μL Bioline dNTPs 2.5 mM each dNTP (part number BIO-39025 BioLine USA, Taunton, MA) and 0.2 μL Roche FastStart™ polymerase 1 Unit (Part number 12 032 945 001 Roche Diagnostic GmbH, Mannheim, Germany) totalling 7.5 μL per 12 μL PCR reaction. PCR was performed in an ABI 9700 thermal cycler under the following conditions: initial denaturation and enzyme activation at 95.0°C for 1 cycle of 6 min, followed by 35 cycles of denaturation at 95.5°C for 55 s, annealing at 58.0°C for 30 s, and extension at 72.0°C for 55 s followed by a final single cycle extension at 72.0°C for 7 min.

### Post PCR processing

After PCR, the Hamilton MicroLab StarPlus added 5 μL of 1× PCR buffer to each well to ensure there was enough volume for the QIAxcel system, and the 384-well plate was distributed to four 96-well plates since the QIAxcel system currently only handles 96-well plates. After visually searching the gel image or spreadsheet output of the QIAxcel system to detect any deletions greater than 14 bp or duplications greater than 14 bp (but less than PCR product size), product concentrations were determined. The Hamilton MicroLab StarPlus condensed the four plates back to a 384-well plate for magnetic bead purification and sequence reaction assembly. PCR purification was per AMPure™ protocol (Agencourt Biosciences Corp. Beverly, MA) slightly modified for the Hamilton robot. Using concentration values from the QIAxcel system in tab delimited format, PCR reactions were selectively diluted by the Hamilton MicroLab StarPlus to achieve normalization of concentration to a range from 2 to 7 ng/μL. Forward and reverse sequence reactions were assembled separately in two 384-well plates. A 1/16^th ^Big Dye™ (Applied Biosystems Inc. part of Life Technologies, Carlsbad, CA) master mix was prepared using: 0.34 μL Big Dye, and 2.41 μL 5× Big Dye Buffer. 2.75 μL master mix was pipetted into the wells of the 384 well sequencing plates which already contained the sequencing primer in 0.5 μL pure glycerol. Next 6.75 μL of the purified and normalized PCR product were pipetted into the appropriate wells. Cycle sequencing was performed on the 10.0 μL sequencing reaction in an ABI 9700 thermal cycler under an initial denaturation at 96.0°C for 4 min followed by 25 cycles of denaturation at 96.0°C for 10 s, annealing at 50.0°C for 5 s, and extension at 60.0°C for 4 min. Sequence reaction purification was accomplished per CleanSeq™ protocol (Agencourt Biosciences Corp. Beverly, MA) also slightly modified for the Hamilton automation. After CleanSeq™, the purified sequencing product plates were heat-sealed and installed in 3730 sequence assembly plates (Applied Biosystems) with Direct Inject Magnets (Agencourt) and loaded into the 3730 sequencer. An ABI 3730 sequencer was used to perform the sequencing operation and Mutation Surveyor and/or SeqScape and/or Sequencher applications were used to analyze the resulting sequences and to search for variations from a consensus sequence.

### Cost Estimates

Table 8 presents an estimate of reagent and plastic costs based on 12 uL PCR and 10 uL sequencing reaction volumes.

One approach to pricing the service would be to average the price per patient over a typical mix of 100 muscular dystrophy patients across all five tiers of testing. Consumable material costs for MLPA (tier 1 = 2 MLPA kits) including plastics and 20% waste are approximately $48.00 ($24.00/kit) and for tier 5 including a much larger number of reference samples is approximately $40.00 per kit. Labor cost is estimated at 2 hours per patient of dedicated technician time for tier 1, 8 hours per patient of one technician for each tier 2-4 - primarily for sample processing in steady state continuous mode - plus 8 hours per patient of a second dedicated technician primarily for sequence analysis, and finally 1 hour per patient per MLPA kit for tier 5. Using those cost estimates, the percentage of DMD incidence (80%, use 60% to be conservative) versus total dystrophies and the percentage of large deletion/duplication (60%) in DMD/BMD cases, and $20.00/hour labor rate, average the direct cost per patient over 100 patients as:

Consumable material costs:

Extract DNA from blood or saliva: 100 × $15.00/patient = $1,500.00

Tier 1 (2-MLPA-rxns.): 100 × $48.00/patient = $4,800.00

Tier 2: 64 non DMD/BMD large del/dup × $532.00 Plate 1 = $34,048.00

Tier 3: 28 remaining undetected × $395.00 Plate 2 = $11,060.00

Tier 4: 18 remaining undetected × $395.00 Plate 3 = $7,110.00

Tier 5: 8 remaining undetected × $40.00 × 4 MLPA's = $1,280.00

Total consumables = $59,798.00/100 = $600.00/patient

Add direct labor:

Extract DNA: 100 patients × 2 hrs./patient × $20.00/hr = $4000.00

Tier 1: 100 patients × 2 hrs./patient × $20.00/hr. = $4,000.00

Tier 2: 64 patients × 16 hrs./patient. × $20.00/hr. = $20,480.00

Tier 3: 28 patients × 16 hrs./patient × $20.00/hr. = $8,960.00

Tier 4: 18 patients × 16 hrs./patient × $20.00/hr. = 5,760.00

Tier 5: 8 patients × 5 hrs./patient × $20.00/hr. = $800.00

Total labor = $44,000.00/100 = $440.00/patient

Total consumables plus labor = $1040.00/patient

Add to this whatever your cost structure requires for indirect/overhead including instrument maintenance costs+ profit + taxes = final price per patient direct cost. Even if that final service pricing cannot be structured this way for various reasons, these numbers still provide a good estimate of the average cost per patient to the department.

Clinicians can significantly decrease the actual cost by steering the molecular testing to a candidate gene based on phenotype, clinical evaluations and any immunolabeling data that has been prepared.

### Quality Goals

Two quality goals were set for assays as follows: 1) No known multiple nucleotide polymorphisms (MNPs) or SNPs within primer sequences except rare SNPs allowed in 5' half of primer. 2) A 12 μL PCR increased to 17 μL with 1×-PCR buffer will have a single sharp band of the planned size and a minimum concentration 0.5 ng/μL (preferably > 2.0 ng/μL) when run on QIAxcel system DNA analyzer (Figure 2).

Three goals were set for gene coverage as follows: 1) All regions of interest will be covered. Regions of interest are pre-defined for each gene and do vary but usually consist of all coding and non-coding exons, splice sites, promoters, 1000 bp of 5' UTR and 3000 bp of 3' UTR. 2) At least 95% of the total sequence for all regions of interest of a given gene is covered by both the sense and anti-sense sequencing reaction. Difficult regions to sequence such as polynucleotide repeats and G/C rich areas will have multiple assays attempting to sequence through the region and will have at least single-stranded coverage. 3) For any given patient, the quality score for every pure (i.e. homozygous) base (as calculated by SeqScape and based on the quality scores of each strand running through that base) for all bases except those in a very few clearly difficult to sequence regions must be greater than 25 on the 0-50 scale used by SeqScape.

### Patient materials and mutation reporting

This study was approved by the Children's Hospital Boston Institutional Review Board. Written informed consent was obtained for each participant. Mutations found using this process should be reported to the Leiden muscular dystrophy pages with technique described as PCR, SEQ.

## List of abbreviations

10×: ten times final concentration; 1×: final concentration; AB and ABI: Applied Biosystems Incorporated (now Life Technologies); BMD: Becker muscular dystrophy; CAPN3: gene symbol for gene that encodes the protein calpain 3; CAV3: gene symbol for gene that encodes the protein caveolin 3; CMD: congenital muscular dystrophy; c.v.: coefficient of variability; DMD: Duchenne muscular dystrophy and its gene symbol; dNTP: deoxynucleotide triphosphate; DYSF: Gene symbol for the gene that encodes the protein dysferlin; Emory: Emory Molecular Genetics Laboratory, Atlanta, Georgia; FKRP: gene symbol for gene that encodes the protein fukatin related protein; INDEL: small DNA insertion or deletion; LGMD: limb-girdle muscular dystrophy; LMNA: gene symbol for gene that encodes the protein lamin A/C; MIM and OMIM: Online Mendelian Inheritance in Man; NCBI: National Center for Biotechnology Information http://www.ncbi.nlm.nih.gov/; PCR: Polymerase Chain Reaction; RefSeq: Reference sequence database in NCBI; SGCA: gene symbol for gene that encodes the protein sarcoglycan alpha; SGCB: gene symbol for gene that encodes the protein sarcoglycan beta; SGCG: gene symbol for gene that encodes the protein sarcoglycan gamma; SGCD: gene symbol for gene that encodes the protein sarcoglycan delta; Tm's: Melting temperature of primers; TRIM32: gene symbol for gene that encodes the protein tripartate containing 32; UTR: untranslated region.

## Authors' contributions

RRB, HES, SB performed sequencing experiments; RRB, HES, ASC, YS, SB, and CB performed sequencing analysis; RRB, HES, PSL, and CB developed automated methods; EE, VL, BLW, PSL, and BTD provided samples and/or patient information; HES, AHB, LMK advised on project design; RRB designed the project, designed the assays, and drafted the manuscript.

## Supplementary Material

Additional file 1 **Alan's table genes**. A table of known myopathy causing genes Click here for file

Additional file 2 **Genetests**. Excel file listing testing services available. Click here for file

Additional file 3 **Calpain 3 v3022108**. Excel file containing CAPN3 assay numbers and associated sequences Click here for file

Additional file 4 **DMD ASSAY LIST Yiping**. Excel file containing DMD assay numbers and associated sequences Click here for file

Additional file 5 **LMNA assay coverage**. Excel file containing LMNA assay numbers and associated sequences Click here for file

Additional file 6 **SGCB assay coverage**. Excel file containing SGCB assay numbers and associated sequences Click here for file

Additional file 7 **SGCG assay coverage09.04.08**. Excel file containing SGCG assay numbers and associated sequences Click here for file

Additional file 8 **CAV3 assay list**. Excel file containing CAV3 assay numbers and associated sequences Click here for file

Additional file 9 **FKRP assay list**. Excel file containing FKRP assay numbers and associated sequences Click here for file

Additional file 10 **SGCA assay coverage**. Excel file containing SGCA assay numbers and associated sequences Click here for file

Additional file 11 **SGCD coverage090808**. Excel file containing SGCD assay numbers and associated sequences Click here for file

Additional file 12 **TRIM32 assay coverage2**. Excel file containing TRIM32 assay numbers and associated sequences Click here for file

Additional file 13 **UCDS_DMD**. A packaged (condensed) methods file for the Hamilton StarPlus robot that can be imported into the Hamilton MicroLab Star Method editor, for the DMD plate (plate 1). This file must be downloaded and saved (not opened) and then imported into the Microlab STAR Method Editor (Hamilton Company). Click here for file

Additional file 14 **UCDS_QUAD**. A packaged (condensed) methods file for the Hamilton StarPlus robot that can be imported into the Hamilton MicroLab Star Method editor, for the Quad plate (plate 2). This file must be downloaded and saved (not opened) and then imported into the Microlab STAR Method Editor (Hamilton Company). Click here for file

Additional file 15 **1116vC-Cav3_Template.pt**. SeqScape™ template file for CAV3. This file must be downloaded and saved (not opened) and the file name must be changed to add .pt at the end of the file name just before the .ctf extension.  For example, change file name from 1471-2156/10/66/suppl/S15.ctf to 1471-2156/10/66/suppl/S15.pt.ctf. The file can then be successfully imported into the SeqScape application (ABI). Click here for file

Additional file 16 **CAPN#-DB.pt**. SeqScape™ template file for CAPN3. This file must be downloaded and saved (not opened) and the file name must be changed to add .pt at the end of the file name just before the .ctf extension.  For example, change file name from 1471-2156/10/66/suppl/S16.ctf to 1471-2156/10/66/suppl/S16.pt.ctf. The file can then be successfully imported into the SeqScape application (ABI). Click here for file

Additional file 17 **DMD_RSS000009308V01_3730_01.pt**. SeqScape™ template file for DMD. This file must be downloaded and saved (not opened) and the file name must be changed to add .pt at the end of the file name just before the .ctf extension.  For example, change file name from 1471-2156/10/66/suppl/S17.ctf to 1471-2156/10/66/suppl/S17.pt.ctf. The file can then be successfully imported into the SeqScape application (ABI). Click here for file

Additional file 18 **FKRP_RSS000009239V01_3730_01.pt**. SeqScape™ template file for FKRP. This file must be downloaded and saved (not opened) and the file name must be changed to add .pt at the end of the file name just before the .ctf extension.  For example, change file name from 1471-2156/10/66/suppl/S18.ctf to 1471-2156/10/66/suppl/S18.pt.ctf. The file can then be successfully imported into the SeqScape application (ABI). Click here for file

Additional file 19 **LMNA.pt**. SeqScape™ template file for LMNAThis file must be downloaded and saved (not opened) and the file name must be changed to add .pt at the end of the file name just before the .ctf extension.  For example, change file name from 1471-2156/10/66/suppl/S19.ctf to 1471-2156/10/66/suppl/S19.pt.ctf. The file can then be successfully imported into the SeqScape application (ABI). Click here for file

Additional file 20 **SGCA_103008.pt**. SeqScape™ template file for SGCA. This file must be downloaded and saved (not opened) and the file name must be changed to add .pt at the end of the file name just before the .ctf extension.  For example, change file name from 1471-2156/10/66/suppl/S20.ctf to 1471-2156/10/66/suppl/S20.pt.ctf. The file can then be successfully imported into the SeqScape application (ABI). Click here for file

Additional file 21 **SGCB_aug_0608.pt**. SeqScape™ template file for SGCB. This file must be downloaded and saved (not opened) and the file name must be changed to add .pt at the end of the file name just before the .ctf extension.  For example, change file name from 1471-2156/10/66/suppl/S21.ctf to 1471-2156/10/66/suppl/S21.pt.ctf. The file can then be successfully imported into the SeqScape application (ABI). Click here for file

Additional file 22 **SGCD1 and D2Copy.pt**. SeqScape™ template file for SGCD. This file must be downloaded and saved (not opened) and the file name must be changed to add .pt at the end of the file name just before the .ctf extension.  For example, change file name from 1471-2156/10/66/suppl/S22.ctf to 1471-2156/10/66/suppl/S22.pt.ctf. The file can then be successfully imported into the SeqScape application (ABI). Click here for file

Additional file 23 **SGCG-Dick051206.pt**. SeqScape™ template file for SGCG. This file must be downloaded and saved (not opened) and the file name must be changed to add .pt at the end of the file name just before the .ctf extension.  For example, change file name from 1471-2156/10/66/suppl/S23.ctf to 1471-2156/10/66/suppl/S23.pt.ctf. The file can then be successfully imported into the SeqScape application (ABI). Click here for file

Additional file 24 **TRIM32.pt**. SeqScape™ template file for TRIM32. This file must be downloaded and saved (not opened) and the file name must be changed to add .pt at the end of the file name just before the .ctf extension.  For example, change file name from 1471-2156/10/66/suppl/S24.ctf to 1471-2156/10/66/suppl/S24.pt.ctf. The file can then be successfully imported into the SeqScape application (ABI). Click here for file

Additional file 25 **UCDSdisply_unk_var_only.ds**. SeqScape™ template file for display settings. This file must be downloaded and saved (not opened) and the file name must be changed to add .ds at the end of the file name just before the .ctf extension.  For example, change file name from 1471-2156/10/66/suppl/S25.ctf to 1471-2156/10/66/suppl/S25.ds.ctf. The file can then be successfully imported into the SeqScape application (ABI). Click here for file

Additional file 26 **UCDSdmddefaults.ad**. SeqScape™ template file for analysis defaults settings. This file must be downloaded and saved (not opened) and the file name must be changed to add .ad at the end of the file name just before the .ctf extension.  For example, change file name from 1471-2156/10/66/suppl/S26.ctf to 1471-2156/10/66/suppl/S26.ad.ctf. The file can then be successfully imported into the SeqScape application (ABI). Click here for file

Additional file 27 **UCDSprotocol1.ap**. SeqScape™ template file for analysis protocol settings. This file must be downloaded and saved (not opened) and the file name must be changed to add .ap at the end of the file name just before the .ctf extension.  For example, change file name from 1471-2156/10/66/suppl/S27.ctf to 1471-2156/10/66/suppl/S27.ap.ctf. The file can then be successfully imported into the SeqScape application (ABI). Click here for file

Additional file 28 **384 welplate tier 2(plate1-DMD)_layout**. Excel file shows the assay locations in DMD plate (plate 1). Click here for file

Additional file 29 **384 welplate tier 3(Plate2)_layout**. Excel file that shows the assay locations in plate 2. Click here for file

Additional file 30 **384 welplate tier 4 (Plate3)_layout**. Excel file that shows the assay locations in plate 3. Click here for file

Additional file 31 **Plate1_assaysbox_EVENSlayout**. Excel file that shows the even DMD assay locations in ScreenMate™ box 1 Click here for file

Additional file 32 **Plate1_assaysbox_ODDSlayout**. Excel file that shows the odd DMD assay locations in ScreenMate™ box 2 Click here for file

Additional file 33 **Plate2_assaysbox_layout**. Excel file that shows the assay locations in ScreenMate™ box for plate 2 Click here for file

Additional file 34 **Plate3_assaysbox_layout**. Excel file that shows the assay locations in ScreenMate™ box for plate 3 Click here for file

Additional file 35 **DMD-conversion384-4x96**. Excel file showing where assays from a DMD plate will end up in four 96-well plates when taken out of the 384-well plate by quads. Click here for file

Additional file 36 **Tips on using SeqScape**. Short tutorial on setting up and using SeqScape. Click here for file

Additional file 37 **DMDT1**. Picture of QIAxcel report DMD assays in 96 well plate A1 for two patients and one no template control. (See additional file 35 for key to assay number.) Click here for file

Additional file 38 **DMDT2**. Picture of QIAxcel report DMD assays in 96 well plate A2 for two patients and one no template control. (See additional file 35 for key to assay number.) Click here for file

Additional file 39 **DMDT3**. Picture of QIAxcel report DMD assays in 96 well plate B1 for two patients and one no template control. (See additional file 35 for key to assay number.) Click here for file

Additional file 40 **DMDT4**. Picture of QIAxcel report DMD assays in 96 well plate B2 for two patients and one no template control. (See additional file 35 for key to assay number.) Click here for file

Additional file 41 **myopathies background, sequence examples and optimization**. Seqscape screenshot figures for two compound heterozygous mutations. Click here for file

Additional file 42 **How to design Assays**. MS Word file describing the process used to design primer assays Click here for file

Additional file 43 **Automation Equipment Evaluation**. List of automated liquid handling system evaluation criteria. Click here for file

Additional file 44 **Process Flow**. Excel file showing a flow diagram of the automated process for the DMD (Plate 1) Click here for file

Additional file 45 **Process Flow Quad**. Excel file showing a flow diagram of the automated process for the Quad (Plate 2 or 3) Click here for file
