# Supplementary material for: Automated DNA mutation detection using universal conditions direct sequencing: application to ten muscular dystrophy genes
Source: BMC Genet. 2009 Oct 18;10:66. doi: 10.1186/1471-2156-10-66 (PMC2781300; doi:10.1186/1471-2156-10-66)
Supplement: Additional file 1 — Alan's table genes. A table of known myopathy causing genes [file 1471-2156-10-66-S1.DOC]

| **Gene Name** | Protein | Phenotype | **DNA testing available?** | **#**  **exons** | **Approx.**  **# UCDS**  **Amplicons** | **UCDS Amplicons verified as**  **of 7/22/08** | **Amplicons in house yet to be verified** |
| --- | --- | --- | --- | --- | --- | --- | --- |
| PLATE #1 |  |  |  |  |  |  |  |
| DMD | dystrophin | Duchenne/Becker | Clinical | 79 | 128 | 128 |  |
|  |  |  |  |  |  |  |  |
| PLATE #2 |  |  |  |  |  |  |  |
| CAV3 | caveolin | LGMD 1C | Research | 2 | 11 | 11 |  |
| ***FKRP*** | fukutin related | LGMD 2I(CMD) | Clinical | 4 | 18 | 18 |  |
| ***CAPN3*** | calpain3 | LGMD 2A | Clinical | 30 | 52 | 52 |  |
| ***TRIM32*** | tripartate contain32 | LGMD 2H | Research | 3 | 15 | 15 |  |
|  |  |  |  |  |  |  |  |
| ***PLATE #3*** |  |  |  |  |  |  |  |
| ***SGCA*** | sarcoglycan alpha | LGMD 2D | Clinical | 10 | 13 | 13 |  |
| ***SGCB*** | sarcoglycan beta | LGMD 2E | Clinical | 6 | 21 | 21 |  |
| ***SGCG*** | sarcoglycan gamma | LGMD 2C | Clinical | 8 | 19 | 19 |  |
| ***SGCD*** | sarcoglycan delta | LGMD 2F | Clinical | 10 | 21 | 21 |  |
| ***LMNA*** | lamin a/c | LGMD 1B  (EDMD) | Clinical | 12 | 15 | 15 |  |

|  |  |  |  |  |  |  |  |
| --- | --- | --- | --- | --- | --- | --- | --- |
| ***PLATE #4*** |  |  |  |  |  |  |  |
| ***DYSF*** | dysferlin | LGMD 2B | Clinical | 55 | 70 | 8 | 28 |
| ***TCAP*** | telethonin | LGMD 2G | No | 16 | 8 |  |  |
| ***TTID*** | myotilin | LGMD 1A | No | 10 | 22 |  |  |
|  |  |  |  |  |  |  |  |
| ***PLATE #5*** |  |  |  |  |  |  |  |
| ***NEB*** | nebulin | Nemaline | Clinical | 183 | 200 |  |  |
|  |  |  |  |  |  |  |  |
| ***FUTURE PLATES*** |  |  |  |  |  |  |  |
| ***ACTA1*** | skeletal actin | Nemaline | Clinical | 7 | 11 |  |  |
| ***FCMD*** | Fukutin | FCMD/LGMD2L | Clinical | 10 | 36 | 20 | 26 |
|  |  |  |  |  |  |  |  |
| ***TPM2*** | beta tropomyosin2 | Nemaline | Clinical |  | 12 |  |  |
| ***TPM3*** | alpha tropomyosin3 | Nemaline | Clinical |  | 26 |  |  |
| ***TNNT1*** | troponin t1 | Nemaline | Clinical |  |  |  |  |
| ***MTN1*** | myotubularin | Myotubular | No | 15 | 28 |  |  |
| ***RYR1*** | ryanodine receptor | CCD/MmD/MH | Clinical |  | 119 |  |  |
| ***EMD*** | emerin | X-EMD(EDMD) | Clinical | 6 | 13 |  |  |
| ***LAMA2*** | lamin a/c | CMD | Clinical | 65 | 92 |  |  |
| ***SGCE*** | sarcoglycan epsilon | MDS(DYT11) | Clinical | 11 | 27 |  |  |
| ***SGCZ*** | sarcoglycan zeta |  | No | 8 | 24 |  |  |
| ***ITGA7*** | integrin a7 | CMD | No | 25 | 40 |  |  |
| ***PABP2*** | polyAbindingp2 | Oculapharyngeal | No |  | 19 |  |  |
| ***SEPN1*** | selenoproteins1 | Rigid Spine | Research | 12 | 27 |  |  |
| ***POMGnT*** | O-mannoseB1,2 | Muscle EyeBrain  LGMD 2M | Clinical | 23 | 42 |  |  |
| ***POMT1*** | O-mannosyl-transferase 1 | Walker-Warberg  LGMD 2K | Clinical | 20 | 31 |  |  |
| ***POMT2*** | O-mannosyl-transferase 2 | LGMD 2N | Clinical |  |  |  |  |
| ***COL6A1*** | colagentypeV1a1 | Ullrich(Bethlem) | No | 35 | 43 |  |  |
| ***COL6A2*** | colagentypeV1a2 | Ullrich(Bethlem) | No | 28 | 40 |  |  |
| COL6A3 | colagentypeV1a3 | Ullrich(Bethlem) | No | 35 | 69 |  |  |
