# Supplementary material for: Automated DNA mutation detection using universal conditions direct sequencing: application to ten muscular dystrophy genes
Source: BMC Genet. 2009 Oct 18;10:66. doi: 10.1186/1471-2156-10-66 (PMC2781300; doi:10.1186/1471-2156-10-66)
Supplement: Additional file 36 — Tips on using SeqScape. Short tutorial on setting up and using SeqScape. [file 1471-2156-10-66-S36.DOC]

Using SeqScape^tm^ software (Applied Biosystems Inc. part of Life Technologies, Carlsbad, CA) a project is created from menu item File/new project, a project name is typed in and a template selected from the list of available templates (Additional files 15,16,17,18,19,20,21,22,23 and 24). Use the UCDS display, analysis and protocol settings from:

### Additional file 25 – UCDSdisply_unk_var_only.ds.ctf

SeqScape^tm^ template file for display settings

### Additional file 26 – UCDSdmddefaults.ad.ctf

SeqScape^tm^ template file for analysis defaults settings

### Additional file 27 – UCDSprotocol1.ap.ctf

SeqScape^tm^ template file for analysis protocol settings

The correct active layer is chosen (It is important to get a layer you like. Hopefully one that has the amino acid sequence correctly associated with coding sections of exons). Samples (patient sequences) are imported to the project from menu item File/import samples to project.

Once sample sequences are loaded, click the green arrow to analyse the specimen. In the project navigator pane, highlight the project name. Then under the “Project view” tab, turn the arrow beside Specimen 1 down. Click on any exon in the line that begins with the active layer name (the active layer and regions of interest line from Figure 2 in the paper) just under the “Project view” tab and then click on any base in the reference sequence. Sequence (if you have any good sequence covering that exon) will appear. Use the TAB and SHIFT TAB keys to search back and forth

to analysing the sample DNA sequence looking for mutations (causative variations from the reference or consensus sequence). Be very careful of situations in which sequence is high quality in one direction up to a certain point and then goes suddenly to poor quality while at the same time sequence is good in the other direction from the other side of that fragment up to nearly the same point and then suddenly goes bad. This is almost always a sure sign of a small deletion, duplication or insertion. If no causative mutation is found using the tab key, visually check all sequence base by base.
