# Supplementary figures and images for: Automated DNA mutation detection using universal conditions direct sequencing: application to ten muscular dystrophy genes
Source: BMC Genet. 2009 Oct 18;10:66. doi: 10.1186/1471-2156-10-66 (PMC2781300; doi:10.1186/1471-2156-10-66)

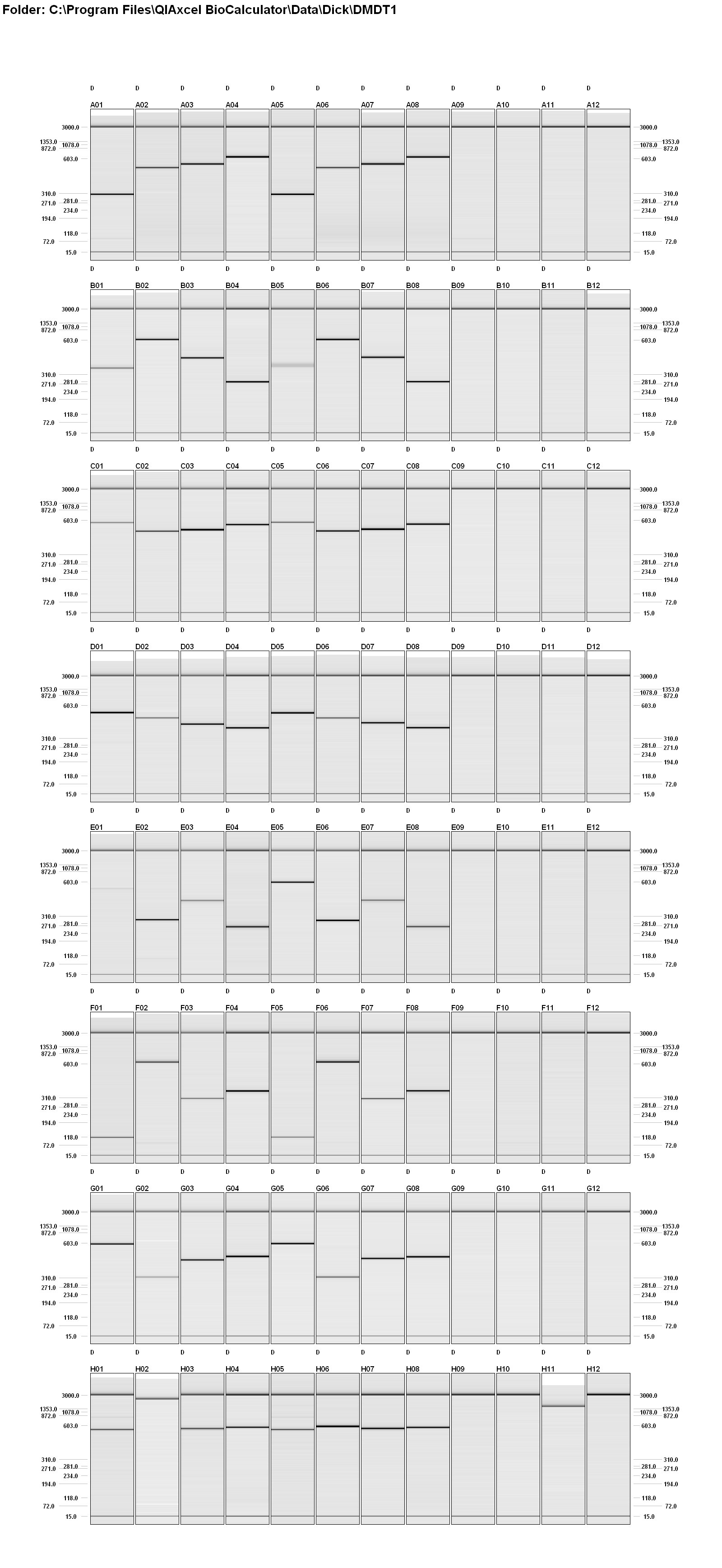

Supplement: Additional file 37 — DMDT1. Picture of QIAxcel report DMD assays in 96 well plate A1 for two patients and one no template control. (See additional file 35 for key to assay number.) [file 1471-2156-10-66-S37.JPEG]

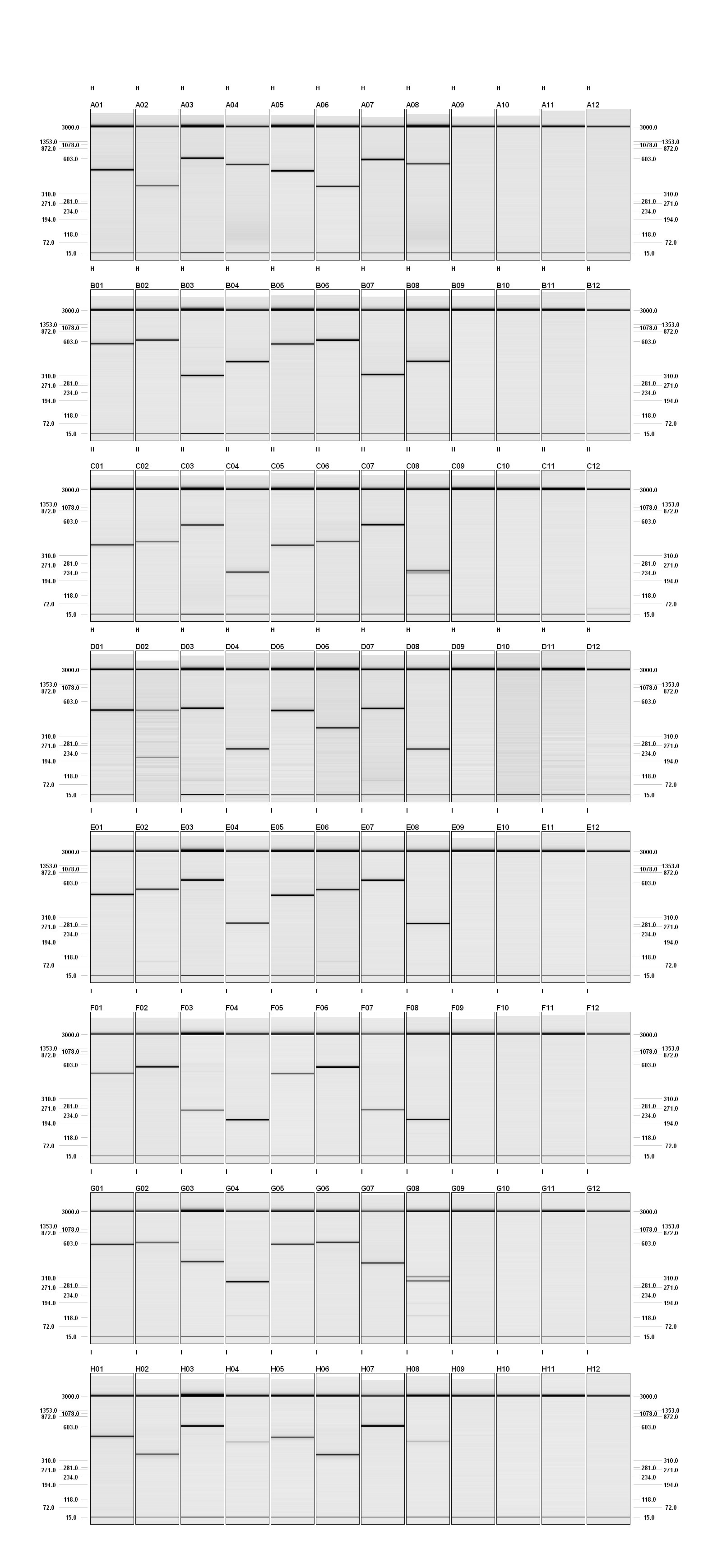

Supplement: Additional file 38 — DMDT2. Picture of QIAxcel report DMD assays in 96 well plate A2 for two patients and one no template control. (See additional file 35 for key to assay number.) [file 1471-2156-10-66-S38.JPEG]

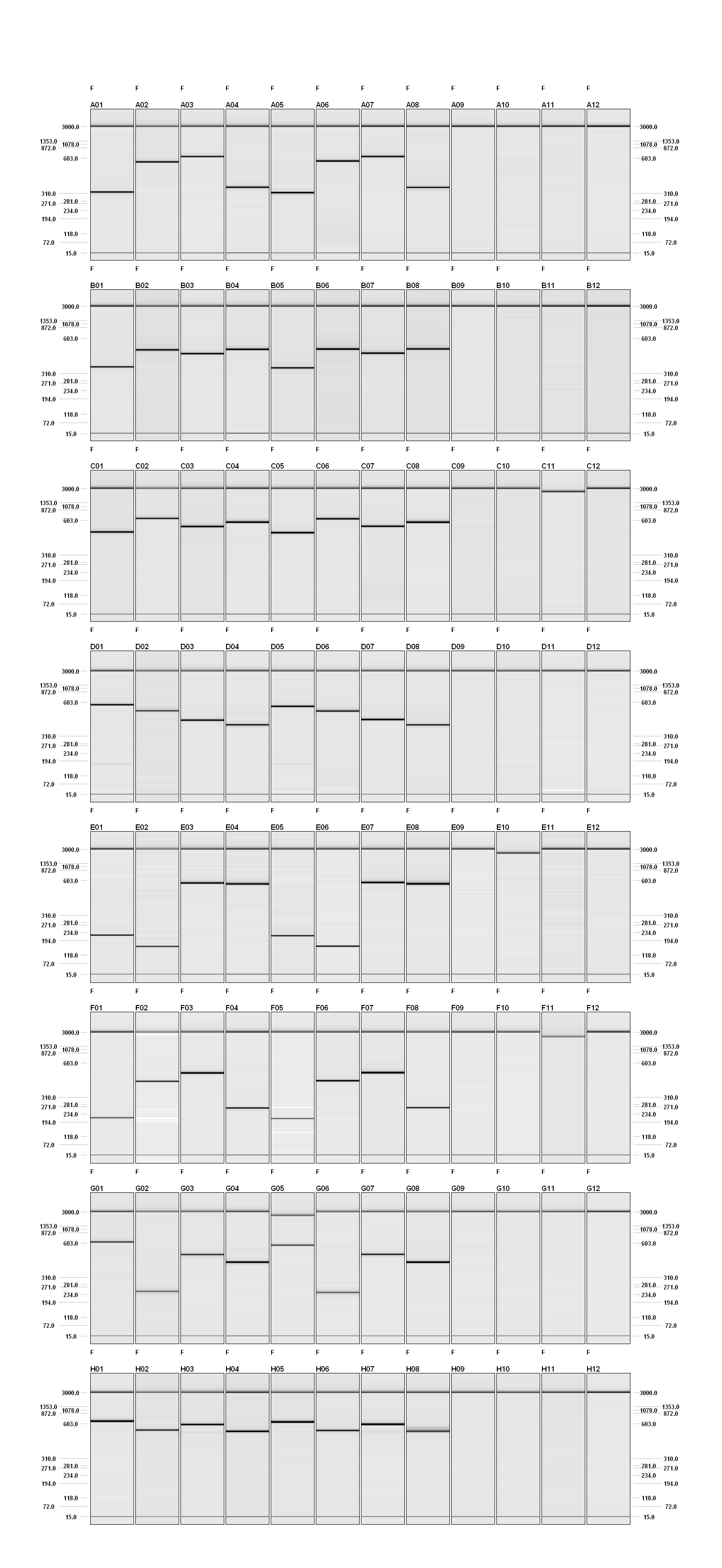

Supplement: Additional file 39 — DMDT3. Picture of QIAxcel report DMD assays in 96 well plate B1 for two patients and one no template control. (See additional file 35 for key to assay number.) [file 1471-2156-10-66-S39.JPEG]

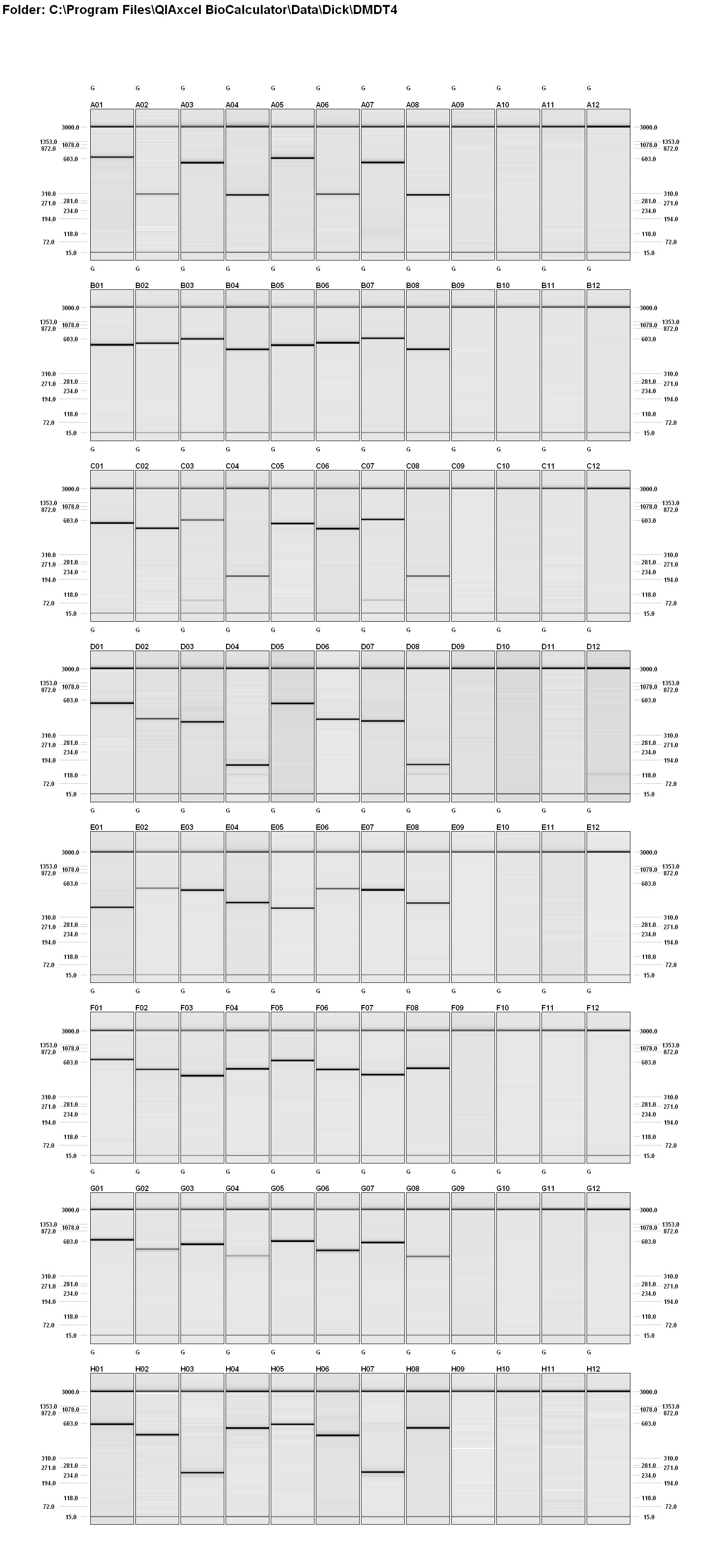

Supplement: Additional file 40 — DMDT4. Picture of QIAxcel report DMD assays in 96 well plate B2 for two patients and one no template control. (See additional file 35 for key to assay number.) [file 1471-2156-10-66-S40.JPEG]
