# Supplementary material for: Automated DNA mutation detection using universal conditions direct sequencing: application to ten muscular dystrophy genes
Source: BMC Genet. 2009 Oct 18;10:66. doi: 10.1186/1471-2156-10-66 (PMC2781300; doi:10.1186/1471-2156-10-66)
Supplement: Additional file 41 — myopathies background, sequence examples and optimization. Seqscape screenshot figures for two compound heterozygous mutations. [file 1471-2156-10-66-S41.DOC]

**Additional/Supplemental File #41:**

**Detailed background on myopathies and sequence examples of heterozygosity detection**

The muscular dystrophies are a subdivision of myopathies (from the Greek for muscle weakness) also known as primary muscle disorders. There are over 40 congenital myopathies. Myopathy is a broad medical term for muscle diseases both congenital and non-congenital. The congenital myopathies can be subdivided into two main categories: dystrophies and other congenital myopathies. The most prominent congenital myopathies are the muscular dystrophies, of which there are 9 types always classified [1] as dystrophies and many more that may be categorized as such. The majority of dystrophies are characterized by progressive muscle degeneration resulting over a varying number of years in near total loss of muscle function. The other congenital myopathies are often referred to in the literature simply as the congenital myopathies or just the myopathies. The majority of these other congenital myopathies are non-progressive or slowly progressive. Other congenital myopathies include myotonia which is characterized by slow muscle relaxation time and many more such as: nemaline myopathy, multiminicore disease, centronuclear myopathy, mitochondrial myopathy, inclusion body myopathy, etc. [2] Many of these diseases are rare. The incidence, defined as the number of new cases per million live births, (or alternatively as the fraction of live births represented by one new case) is not known for many of the more rare diseases. These primary muscle disorders, however, can be subdivided into four groups in which estimates of incidence based on published data can be made.

Table 1 – Incidence data for four subdivisions of congenital myopathies

|  | Maximum incidence | | Minimum incidence | | Average | |
| --- | --- | --- | --- | --- | --- | --- |
| Type | freq. x10^-6^ | fraction | freq. x10^-6^ | fraction | x10^-6^ | fraction |
| DMD | 300 | 1/3333 | 169 | 1/5917 | 235 | 1/4255 |
| BMD | 54 | 1/18500 | 17 | 1/58800 | 35 | 1/28571 |
| Other  dystrophies | 100 | 1/10000 | 41 | 1/24390 | 70 | 1/14286 |
| myopathies | 219 | 1/4566 | 100 | 1/10000 | 160 | 1/6250 |
| Total | 673 | 1/1486 | 327 | 1/3050 | 500 | 1/2000 |

**Table 1:** DMD mean incidence is well established at 300x10^-6^ (Emery 2002) [3] but data varies from country to country and survey to survey. It is unlikely to be much higher but may be as low as 169 x10^-6^ (Cowan 1980).[4] BMD frequency is based on (Emery 1991).[5] Thankfully, both DMD and BMD incidence is probably declining annually due to genetic testing and counselling.Other dystrophy incidence numbers are scarce to non-existent. The rough estimate maximum is extrapolated from (Bushby 2001) [6] and minimum from (Emery 2002) [3] Other congenital myopathy numbers are also scarce to non-existent. The maximum estimate is extrapolated from Nonaka 2001 [7] as 81% of the mean DMD incidence. The minimum estimate is extrapolated from (D’Amico 2008) [8] and (Lopate 2007) [9]. These data were used for estimation of testing cost and should be considered as rough estimates only.

## Figure 1 - Patient 1092.1 heterozygous mutations found in CAPN3 gene


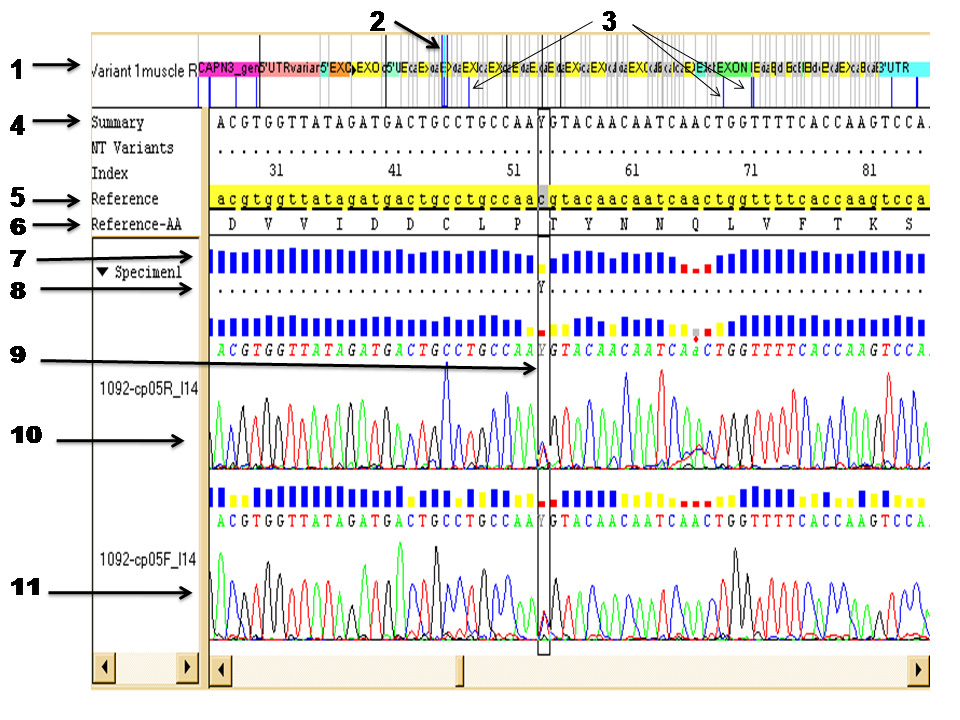


**Figure 1.** Seqscape image capture A) CAPN3 assay cp05 upper (forward) and lower (reverse) sequence. 1) active layer regions of interest, 2) location of cursor in active layer, 3) location of variations from reference sequence, 4) summary sequence showing ambiguities, 5) reference nucleotide sequence, 6) reference amino acid sequence 7) quality bars, 8) ambiguities, 9) location of cursor in sequence at heterozygous mutation c551 C>T, patient 1092.1, 10) assay cp05R patient 1092.1, 11) assay cp05F patient 1092.1

## Figure 2 - Patient 1092.1 probable compound heterozygous mutations CAPN3 gene


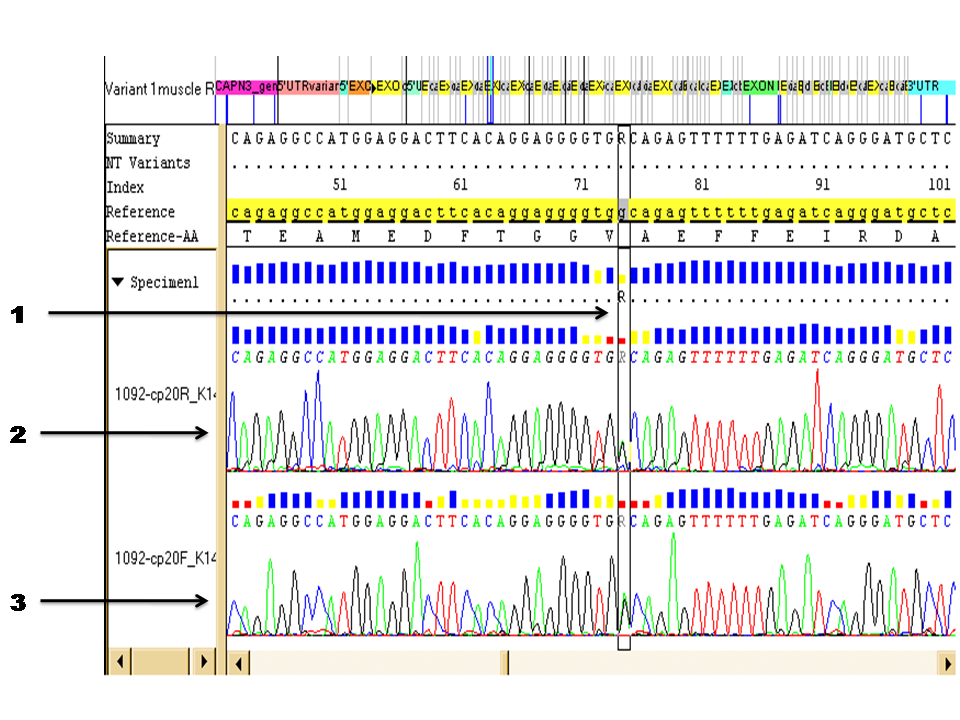


**Figure 2.** CAPN3 assay cp20 upper (forward) and lower (reverse) sequence, 1) location of cursor at probable compound heterozygous mutation c706G>A, 2) assay cp20R patient 1092.1, 3) assay cp20F patient 1092.1

## Figure 3 - Patient 1102.1 probable heterozygous deletion mutation CAPN3 gene


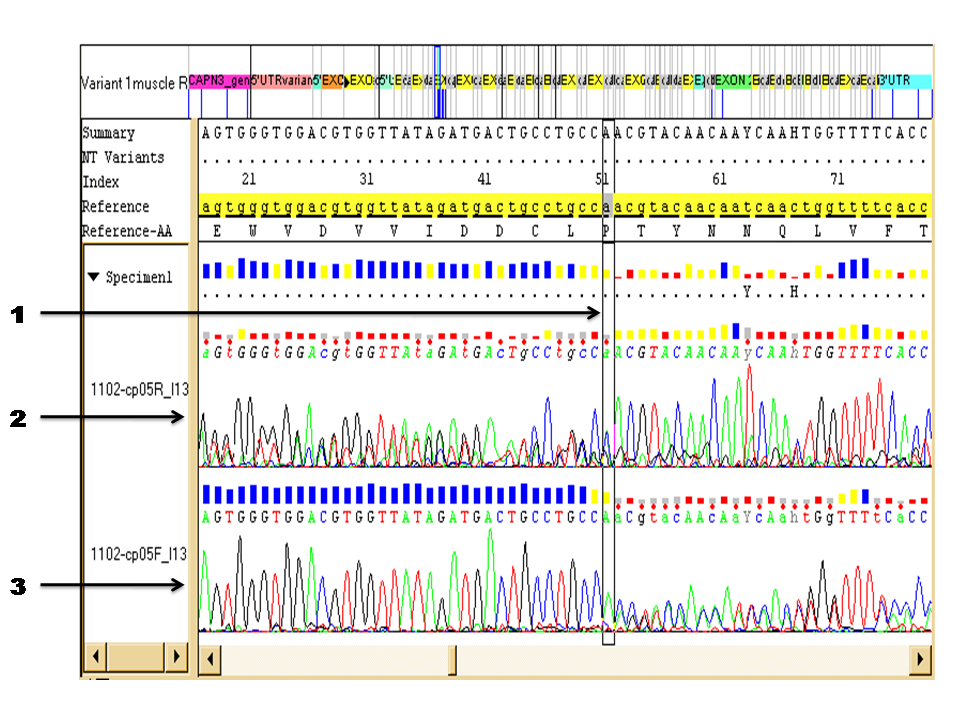


**Figure 3.** CAPN3 assay cp05 upper (forward) and lower (reverse) sequence, 1) location of cursor at heterozygous mutation c550delA, 2) assay cp05R patient 1102.1, 3) assay cp05F patient 1102.1

**Figure 4 - Patient 1102.1 probable compound heterozygous duplication mutation CAPN3 gene**


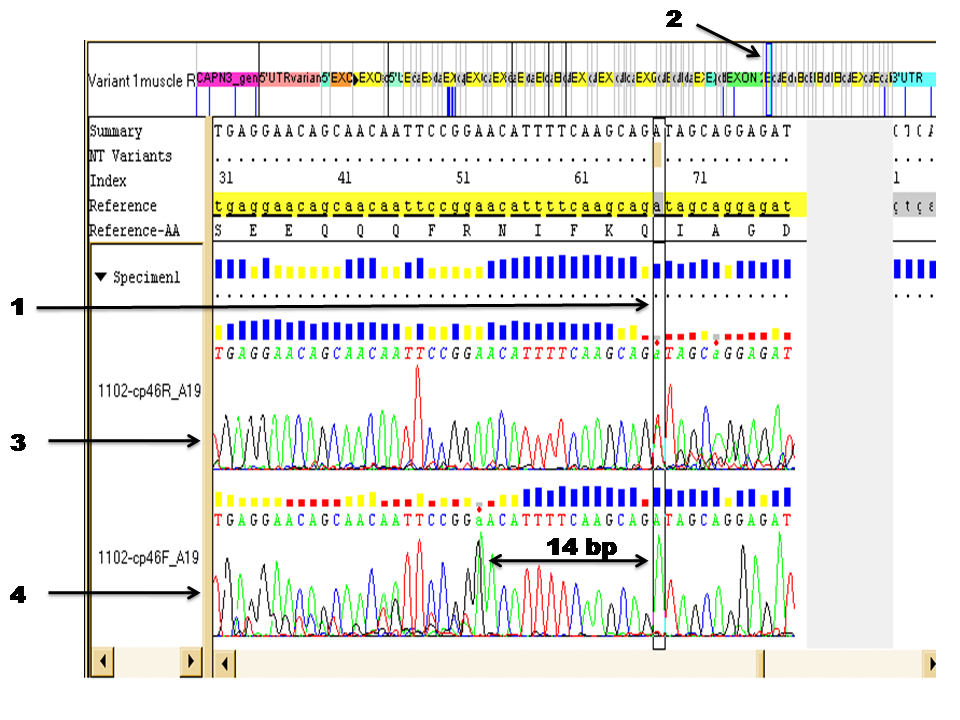


**Figure 4.** CAPN3 assay cp46 upper (forward) and lower (reverse) sequence, 1) location of cursor at probable compound heterozygous mutation c1967dupACATTTTCAAGCAG, 2) location of cursor, Note there is no blue variation from consensus marker here, 3) assay cp46R patient 1102.1, 4) assay cp46F patient 1102.1

**Flexibility and potential future optimizing of the process**

The process is flexible enough to allow for process improvements as automation, PCR and sequencing technologies improve. For example, possible optimizations include: Change PCR and cycle sequencing polymerase to a more universally efficient system; reduce PCR volume; reduce cycle sequencing reaction volume; replace universal sequencing tails M13F and M13R with P172 (5'-TATAGGGCGAATTGGGTAC – 3' ) and P79 (5'-GGTGGCGGCCGCTCTAG – 3' ) or other more matched and universally efficient primers; switch from 384-well to 1536-well plates; replace the QIAxcel system with a more accurate and efficient system of PCR concentration and size measurement including 384-well plates and better detection of heterozygous deletions and duplications down to less than 14 base pairs; (possibly Lab 90; Caliper systems, Hopkington, MA); reduce the relative amounts of PCR and cycle sequencing polymerase and primers in these reactions; add 1M Betaine and/or 5%DMSO to the PCR reactions; replace CleanSeq sequencing reaction clean up system with a more efficient technique (possibly Edge Bio AutoDTR384; Edge BioSystems, Gaithersberg, MD) replace less efficient assays with more efficient assays; design assays for more genes.

**An appropriate quotation**

As expressed by Judith Cowan, “It has been argued that such precision of diagnosis is a luxury. For patients within this group, however, it is not a luxury but rather a right which dictates, in many cases, future life choices. Understanding why a disease has happened, where it has come from, what it is likely to cause in the future and what the implications are for one’s own children and for relatives in the broader family - all these answers come with precise diagnosis and are all absolutely critical questions in the evolution of a patient coming to terms with one of these chronically disabling disorders.”[4]
