# Supplementary material for: Automated DNA mutation detection using universal conditions direct sequencing: application to ten muscular dystrophy genes
Source: BMC Genet. 2009 Oct 18;10:66. doi: 10.1186/1471-2156-10-66 (PMC2781300; doi:10.1186/1471-2156-10-66)
Supplement: Additional file 42 — How to design Assays. MS Word file describing the process used to design primer assays [file 1471-2156-10-66-S42.DOC]

**Assay Design**

Definitions:

An assay is defined for our purposes as consisting of a forward and a reverse primer in the same solution. Assay design begins with template design.

A template is part of the analysis software used to identify mutations and is based on reference sequence from NCBI. We use either Mutation Surveyor^tm^ (SoftGenetics, LLC. State College, PA), Seqscape^tm^ (Applied Biosystems Inc. part of Life Technologies, Carlsbad, CA), Sequencher ^tm^ (Gene Codes Inc., Ann Arbor, MI) as our sequence analysis software.

Here is the process we use:

We begin designing a template for Seqscape (or Surveyor) by accessing the Ensembl database. [www.ensembl.org](http://www.ensembl.org/). ensembl is a joint project between EMBL-EBI (European Molecular Biology Laboratory-European Bioinformatics Institute and the Sanger institute. Search the genome for the gene of interest and print out the genomic sequence including sufficient 5’ and 3’UTR (usually about 3000bp is more than enough) to include the most important promoters and the poly A signal.). Use one color for exons and another color SNPs. Identify and highlight coding and non-coding sections of exons, promoters, isoforms, and poly A signal using cDNA, protein and other databases such as [www.DMD.nl](http://www.DMD.nl/). From this effort you will have one or more color coded print outs of the genomic sequence on which to map your designed assays. Here you can check your designs to eliminate unwanted Simple Nucleotide Polymorphisms including Single Nucleotide Polymorphisms, insertions duplications deletions deletions plus insetions ( indels) etc..

The second step in creating a template for SeqScape is to access The NCBI (National Center for Biotecnology Information) database at <http://www.ncbi.nlm.nih.gov/> and from the index list on the left column of the home page chose GenBank and in the GenBank menu bar chose Entrez (the Life Sciences Search Engine) and chose the Nucleotide database. Search Nucleotide for your gene of interest, select the gene (in the correct genome) and scroll down to the Reference assembly Genomic subheading and click on download genbank under NT_xxxxxxxx or AC_xxxxxxx. In the menu boxes at the top of the page underneath the Search (Nucleotide) box is a set of three boxes in a row. Display (GenBank), Show (5) a third box. In that third box, select Send to, and then again select in the same box (File). You will get a newly opened window labled “opening sequences.gb” with defaults “open with notepad” and radio button “save file”.

Just click o.k. On your desktop, a file will appear which has the sequence in .gb format which can be loaded into SeqScape as a template. You may edit this sequence before you load it into SeqScape to add more sequence to the 5’ or 3’ end but you must be very careful to keep exactly the same .gb formating.

Now that you have a .gb file, you can import it into Seqscape (or Surveyor or Sequencher) and go through the process of identifying regions of interest as non-coding portions of exons and coding portions (with translation) of exons etc. in separated layers for each isoform of the gene. Once you have your template in SeqScape and your color coded printout of the genomic sequence including SNPs you are ready to begin designing assays. We use Oligo^tm^ from Molecular Biology Insights Inc., as our primary tool to design primers. We use Sequencher from Gene Codes Inc., SeqScape with template and our color coded genomic sequence print out and NCBI BLAST and /tools/primer-BLAST to assist in this process. Assays were designed to assure all would amplify specific regions of DNA at 58 degrees C., without common SNPs anywhere in the primer and without rare SNPs in the 3’ half of the sequence specific primer. For best sequencing results, assays should be designed for approximately 600 bp amplicons even if the region of interest to be sequenced is much smaller than 600 bp to minimize the amount of PCR product concentration normalization necessary when making sequencing reactions. Amplicons must include at least 60 bp on either side of an exon to insure good sequence through splice sites. The above guidelines are not always possible however because primers should be placed between polynucleotides string greater than 7 or 8 bp or clusters of G’s and C’s greater than 5 or 6 within any 8 or 9 bp and the start of the area of interest (exon) because these sequence features do not sequence well. Therefore, it is an iterative process to get the best coverage of any region of interest. It is also beneficial to try and include common SNPs anywhere within the amplicon except in sequence to which primers must bind. This will sometimes assist the analyst in determining heterozygosity and loss of heterozygosity. Here is a list of Primer design criteria:

1] Primers must all anneal at nearly the same temperature (58-60 C).

2] Must all have 60-80 bp on either side of small (<400 bp) exons. This is to ensure both good sequence and capture of splice site mutations.

3] Ideally, all amplicons will be near 600 bp regardless of size of exons.

4] Large exons, polynucleotide strings greater than 7 bases or GC rich clusters greater than 5 bases which do not sequence well will have overlapping amplicons.

5] There will be no Simple Nucleotide Polymorphisms in the target sequence at the primer locations except that very rare polymorphisms may be allowed in the 5 prime half of primer sequences (because they will probably still bind ok even in the rare individuals).

6] Ideally, amplicons will include one or more fairly common polymorphisms somewhere within the amplicon sequence (between the primers, but not in the primers). This will help in establishing heterozygosity of alleles.

7] Of course, no hairpins or primer dimers greater than three bases.

(actually delta G of less (more positive) than -3.0 in 3 prime hairpins and less than -20 for internal dimers.

8] Very specific Blast results.

9] Must have have acceptable internal and 3' stability. That is to say, a strong binding area (GC rich) somewhere in the first 2/3 of the primer and a weak 3' (AT rich) end of the primer.

Of course there will always have to be some trade offs.

In the oligo program, set the “non-search parameters” as follows:

**concentrations**

monovalet cations: 15mM

free Mg[2+]: 1.0mM (because dNTPs chelate half the 2.0mM MgCl_2_)

nucleaic acid concentration: 8000000pm

temperature for delta G 25C

calculations:

Then load the same sequence that you have loaded into the template of SeqScape.

Under Search menu select “for primers and probes” and set up ranges and parameters to search for your first assay. Once you have at least on pair of primers to evaluate in a pop up window, select one and see the PCR window open (or under the analyze menu, chose PCR). In the concentrations box in that window, make changes as follows:

**concentrations**

upper primer: 400nM

lower primer: 400nM

monovalet cations: 15mM

free Mg[2+]: 1.0mM

These values should then re-appear any time you open the PCR window and are used by OLIGO to calculate optimum Ta and many other values.

Iterate your searches varying length, position and whether matching Tm’s or PE’s until all your assays have optimum Ta > 55C and meet all quality goals. Use 58C as the actual anneal temperature for all PCR reactions. Under the analyze menu, you can check for dimers, hairpins, internal stability and false priming sites (use greater than PE=170 as a threshold). Internal stability should be kept below 9 (preferably below 7) at the three prime end of the primer.

Within OLIGO, you can open a database to store and retrieve you assays.

Once you have decided on a pair of primers using OLIGO, go to NCBI primer-BLAST (<http://www.ncbi.nlm.nih.gov/>tools/primer-blast), or just BLAST and test one primer at a time to be sure your chosen pair does not bind at other places in the genome that could result in additional bands.

**Quality Goals**

We have set two quality goals for UCDS assays as follows:

1) No known SNPs within primer sequences except rare SNPs allowed in 5’ half of primer.

2) A 12ul PCR purified by magnetic bead (Ampure) and increased to 17ul with 1X-PCR buffer will have a single sharp band of the planned size and a minimum concentration of 0.5 ng/ul when run on e-Gene’s HDA-GT12 DNA analyzer. See Figure 1 CAV3 UCDS assays.

We have set three goals for UCDS gene coverage as follows:

1) All regions of interest will be covered. Regions of interest are pre-defined for each gene and do vary but usually consist of all coding and non-coding exons, splice sites, promoters, 1000bp of 5’ UTR and 3000bp of 3’ UTR.

2) At least 95% of the total sequence for all regions of interest of a given gene is covered by both the sense and anti-sense sequencing reaction. Difficult regions to sequence such as single and poly nucleotide repeats and G/C rich areas will have multiple assays attempting to sequence through the region and will have at least single stranded coverage.

3) For a given patient the quality score for every pure (i.e. homozygous) base (as calculated by SeqScape and based on the quality scores of each strand running through that base) for all bases except those in a very few clearly difficult to sequence regions must be greater than 25 on the 0-50 scale used by SeqScape.

Designing assays that all amplify at the same anneal temperature and meet the quality goals above is a very iterative process, but well worth the effort.
