# Supplementary material for: Automated DNA mutation detection using universal conditions direct sequencing: application to ten muscular dystrophy genes
Source: BMC Genet. 2009 Oct 18;10:66. doi: 10.1186/1471-2156-10-66 (PMC2781300; doi:10.1186/1471-2156-10-66)
Supplement: Additional file 43 — Automation Equipment Evaluation. List of automated liquid handling system evaluation criteria. [file 1471-2156-10-66-S43.DOC]

**Additional/Supplemental file to UCDS manuscript**

**Evaluation of Automation alternatives:**

Many alternatives exist for automating the UCDS process and the choice of the proper equipment is crucial to successful implementation. An instrument that loads the 384-well PCR plates, normalizes the amounts of PCR product to be used in sequencing reactions and prepares and subsequently purifies sequencing reactions must be nearly perfect and is by far the most important piece of automation equipment. Of course the thermal cycler and sequencer are of fundamental importance and must also be chosen carefully, but these technologies are more mature. To address the importance of the automation choice, we listed a set of requirements for instrument selection as follows:

1. Instrument must have at least 8 tips which can be addressed individually and can aspirate different amounts each and dispense different amounts each.
2. Ideally, the instrument must be able to aspirate as little as 0.5 μL from as little as 2.0 μL at the bottom of wells in a 384-well plate. In practice, even if specified better, we don’t trust aspirating or dispensing less than 2 uL.
3. Robot must be able to dispense as little as 0.5 μL into the wells of a 384-well plate and either dispense into as little as 2.0 μL liquid at the bottom of the well or to dispense against the sides of the well in four directions with a c.v. (coefficient of variability) of less than 10% over 384-wells and a repeatability of 99.8% over at least 1000 repetitions. (Again, this would be ideal.)
4. Multi-dispense mode is often not covered by the c.v. specification of an automated instrument, only single dispense is covered. This is a critical parameter and buyers of automation must beware, test this function and try to get it specified.
5. Ideally, the instrument will also have a 96 or 384 tip head as well as the 8 or more individual tips.
6. Must have disposable tips and a wash station with multiple wash reagent capabilities.
7. Must of course have very accurate x, y, z positioning and sufficient deck layout positions for all reagents and plates necessary.

We have been able to write satisfactory methods for the UCDS process for the Hamilton StarPlus instrument.
